# PDK4 promotes vascular calcification by interfering with autophagic activity and metabolic reprogramming

**DOI:** 10.1038/s41419-020-03162-w

**Published:** 2020-11-17

**Authors:** Wen-Qi Ma, Xue-Jiao Sun, Yi Zhu, Nai-Feng Liu

**Affiliations:** Department of Cardiology, Zhongda Hospital, School of Medicine, Southeast University, 87 Dingjiaqiao, Nanjing, 210009 P.R. China

**Keywords:** Calcium signalling, Calcification

## Abstract

Pyruvate dehydrogenase kinase 4 (PDK4) is an important mitochondrial matrix enzyme in cellular energy regulation. Previous studies suggested that PDK4 is increased in the calcified vessels of patients with atherosclerosis and is closely associated with mitochondrial function, but the precise regulatory mechanisms remain largely unknown. This study aims to investigate the role of PDK4 in vascular calcification and the molecular mechanisms involved. Using a variety of complementary techniques, we found impaired autophagic activity in the process of vascular smooth muscle cells (VSMCs) calcification, whereas knocking down PDK4 had the opposite effect. PDK4 drives the metabolic reprogramming of VSMCs towards a Warburg effect, and the inhibition of PDK4 abrogates VSMCs calcification. Mechanistically, PDK4 disturbs the integrity of the mitochondria-associated endoplasmic reticulum membrane, concomitantly impairing mitochondrial respiratory capacity, which contributes to a decrease in lysosomal degradation by inhibiting the V-ATPase and lactate dehydrogenase B interaction. PDK4 also inhibits the nuclear translocation of the transcription factor EB, thus inhibiting lysosomal function. These changes result in the interruption of autophagic flux, which accelerates calcium deposition in VSMCs. In addition, glycolysis serves as a metabolic adaptation to improve VSMCs oxidative stress resistance, whereas inhibition of glycolysis by 2-deoxy-D-glucose induces the apoptosis of VSMCs and increases the calcium deposition in VSMCs. Our results suggest that PDK4 plays a key role in vascular calcification through autophagy inhibition and metabolic reprogramming.

## Introduction

Vascular calcification is a prevalent complication that occurs more often in patients with atherosclerosis, chronic kidney disease, hypertension, or diabetes mellitus or who are ageing or smoking^1,2^. At pathogenic and clinical levels, vascular calcification shares many similarities with bone formation, and its severity is commonly accompanied by a heightened risk of cardiovascular events and all-cause mortality; however, no therapeutics are currently available. Multiple cardiovascular-related cells, including endothelial cells, pericytes, vascular smooth muscle cells (VSMCs) and valve interstitial cells, are involved in vascular calcification, and among these, VSMCs are the predominant cell type involved, having biosynthetic, proliferative and contractile roles in the vessel wall^3,4^. Continuous intracellular or environmental stimuli, including oxidative stress, hypoxia, abnormal mineral metabolism, apoptosis and chronic inflammation, contribute to a phenotypic change of VSMCs to osteoblast-like cells, in which master transcription factors of osteogenesis and chondrogenesis are upregulated concomitant with the generation of mineralized extracellular matrix; parallel with this process, VSMCs lose a range of contractile proteins, eventually causing vascular calcification^5^. Under basal conditions, VSMCs display higher rates of glucose metabolism and lactate production than do other normal cells^6^, despite oxidative phosphorylation is the main site for the production of ATP in these mammalian cells. Furthermore, VSMCs phenotypic changes are tightly integrated with the overall energetic state of the cells, as evidenced during VSMCs proliferation in atherosclerosis^7^ and pulmonary arterial hypertension^8^. Recently, our laboratory found that mitochondrial dysfunction, especially abnormal mitochondrial biogenesis, is associated with the phenotypic switch of VSMCs^9^. These observations indicate that cellular energy metabolic regulation is at the centre of vascular calcification, but the precise regulatory mechanisms remain largely uncertain.

Autophagy is an evolutionarily conserved pathway for the degradation of cellular components through lysosomes, and it is also required for cells that do not have sufficient energy to survive^10^. Autophagy is commonly regarded as a protective mechanism against vascular calcification^11^, but its activity in this role is controversial. Several studies have suggested an increase in autophagic flux and the formation of autophagic vesicles during the calcification process^11^, whereas other investigators have identified that autophagic flux is defective^4,12–14^. These discrepancies may be caused by differences in cell types, assay conditions, or methods to measure autophagy. Therefore, it is necessary to perform a comprehensive evaluation of autophagy. Transcription factor EB (TFEB) was recently discovered as a master regulator of lysosomal biogenesis and autophagy^15^. Once activated, TFEB is translocated into the nucleus to induce the transcription of target genes^16^. In response to the enhanced cellular clearance capability via autophagy, TFEB coordinates an efficient transcription programme to upregulate genes associated with either the early (autophagosome formation) or late (lysosome biogenesis) phases of autophagy^17^. Recent evidence suggests that activation of TFEB alleviates atherosclerosis development in mice by promoting lysosomal biogenesis and autophagy induction^18^. Although atherosclerosis is a chronic inflammatory and age-related disease that shares several common pathogenic mechanisms with vascular calcification, there has been no direct evidence or precise regulatory mechanisms discovered to determine whether TFEB and autophagy cooperate in the regulation of vascular calcification.

Pyruvate dehydrogenase kinases (PDKs) are key enzymes in mitochondria that negatively regulate the activity of the pyruvate dehydrogenase (PDH) complex through the phosphorylation of its subunit, thus contributing to the regulation of glucose utilization and lipid metabolism^19,20^. Alterations in PDK expression and activity contribute to a variety of physiological and disease-linked processes, including insulin resistance and diabetes mellitus, cancer and obesity^21^. Four PDK isoenzymes are known to be expressed in a tissue-specific manner in mammals, with PDK4 being the predominant isoform in tissues with high energy demands, such as skeletal muscle, heart, lactating mammary gland, liver and vascular tissue^19,22^. Clinical data indicated that PDK4 is upregulated in the calcified vessels of patients with atherosclerosis^22^. Mice deficient for PDK4 in VSMCs exhibit a significant reduction in aortic calcium accumulation after vitamin D3 treatment compared with this accumulation in the control mice^22^. In ApoE^−/−^ mice, inhibition of PDK4 by dichloroacetate (DCA), a well-established PDK inhibitor, attenuates atherosclerosis^23^. Recent studies from our laboratory and others have found that PDK4 activity is closely related to mitochondrial function in VSMCs^9,22^; however, the exact mechanisms by which it affects pathological processes are poorly understood. New evidence suggests that PDK4 is a novel modulator of the integrity of the mitochondria-associated ER membrane (MAM), a structural link between the endoplasmic reticulum (ER) surface and the outer mitochondrial membrane that acts as crucial signalling hub in cellular energy homoeostasis, ER stress, mitochondrial injury, autophagy, apoptosis, inflammation and calcium homoeostasis^24–26^. This evidence prompted us to investigate whether the regulation of PDK4 functions in mitochondria are associated with alterations in ER-mitochondrial interactions in the context of vascular calcification.

In light of previous observations, the goal of the current work was to determine how PDK4 affects mitochondrial metabolism in VSMCs and to identify whether such changes regulate the VSMCs phenotypic switch and vascular calcification. In addition, we also investigated the correlation between PDK4 and autophagy. We observed that PDK4 could lead to mitochondrial fragmentation and disruption of the integrity of the MAM, which might result in a decrease in mitochondrial respiratory capacity. Moreover, PDK4 inhibits the nuclear translocation of TFEB, thus inhibiting lysosomal function. These changes result in decreased autophagic activity, which has been implicated in the pathogenesis of vascular calcification. In addition, we found that PDK4 drives the metabolic reprogramming of VSMCs towards a Warburg effect and that lactate, a by-product of glycolysis, plays a protective role to improve VSMCs resistance to oxidative stress under a calcification microenvironment.

## Results

### PDK4 expression is upregulated during both vitamin D3 plus nicotine-induced aortic calcification in rats and β-GP-induced calcification of VSMCs

**Fig. 1:**
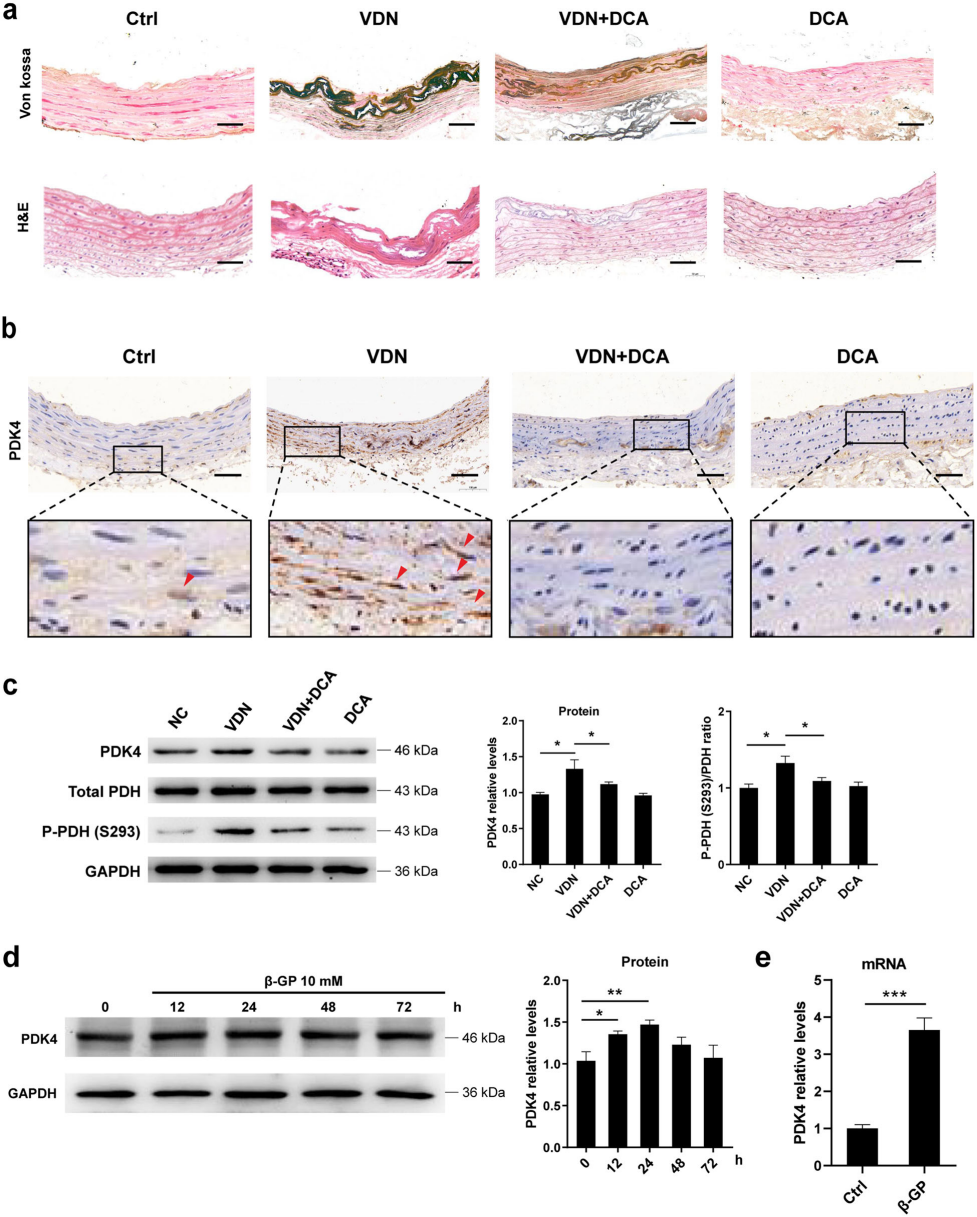
PDK4 is upregulated during the vascular calcification. **a** Representative Von Kossa staining and H&E staining in aortic tissues isolated from SD rats (original magnification, ×200; Scale bars = 100 μm). *N* = 6 rats per group. **b** Representative immunohistochemical staining of PDK4 in aortic tissues isolated from SD rats (original magnification, ×200; Scale bars = 100 μm). *N* = 6 rats per group. **c** Protein expression of PDK4, phospho-PDH (S293), and PDH was determined by western blotting. *N* = 4–6 rats per group. **d** VSMCs were exposed to 10 mM β-GP for 0–72 h, and the protein expression of PDK4 was determined using western blotting. *N* = 3 independent experiments. **e** VSMCs were treated with 10 mM β-GP for 24 h. RT-qPCR analysis was used to detect the mRNA level of PDK4 in the VSMCs. *N* = 3 independent experiments. *P < 0.05, **P < 0.01, ***P < 0.001.

An animal model of arterial calcification was established by the administration of vitamin D3 plus nicotine (VDN) in Sprague-Dawley (SD) rats. Histological assessments with H&E staining showed that the VSMCs were clear and arranged neatly in the negative control (NC) group, while disordered smooth muscle arrangement and vacuolization were observed in the aortic tissues of the VDN group (Fig. 1a). Von Kossa staining showed a significant increase in calcium deposition in the VSMCs and extracellular matrix of aortic tissues compared with that in the NC group (Fig. 1a). To elucidate the role of PDK4 in vascular calcification, we first determined the levels of PDK4 protein expression in calcified aortic tissues. Immunohistochemistry results showed the positive immunostaining for PDK4 in calcified aortic tissues of VDN group, with weak immunostaining in the aortic tissues of NC and VDN + DCA groups (Fig. 1b). Furthermore, the protein expression of PDK4 was increased in the VDN group compared with its expression in the NC group, while DCA treatment suppressed its protein levels (Fig. 1c). In addition, we investigated the phosphorylation level of PDH to indirectly evaluate PDK4 activity. The phospho-PDH (S293)/PDH ratio was increased in calcified aortic tissues of VDN group compared with the NC group, which reflects that enhanced PDK activity likely results from increased PDK4 expression during the calcification process (Fig. 1c).

To mimic vascular calcification in vitro, cells were subjected to calcifying medium containing β-GP. The protein expression of PDK4 in β-GP-treated VSMCs was significantly increased at 12 h and then was decreased at 48 h and 72 h (Fig. 1d). Consistently, the mRNA level of PDK4 was also increased upon β-GP treatment (Fig. 1e). Taken together, these results suggest increased PDK4 activity and protein expression under calcification conditions.

### PDK4 promotes VSMCs phenotypic switching and calcium deposition, which is accompanied by mitochondrial fragmentation and metabolic reprogramming in the calcification microenvironment

**Fig. 2:**
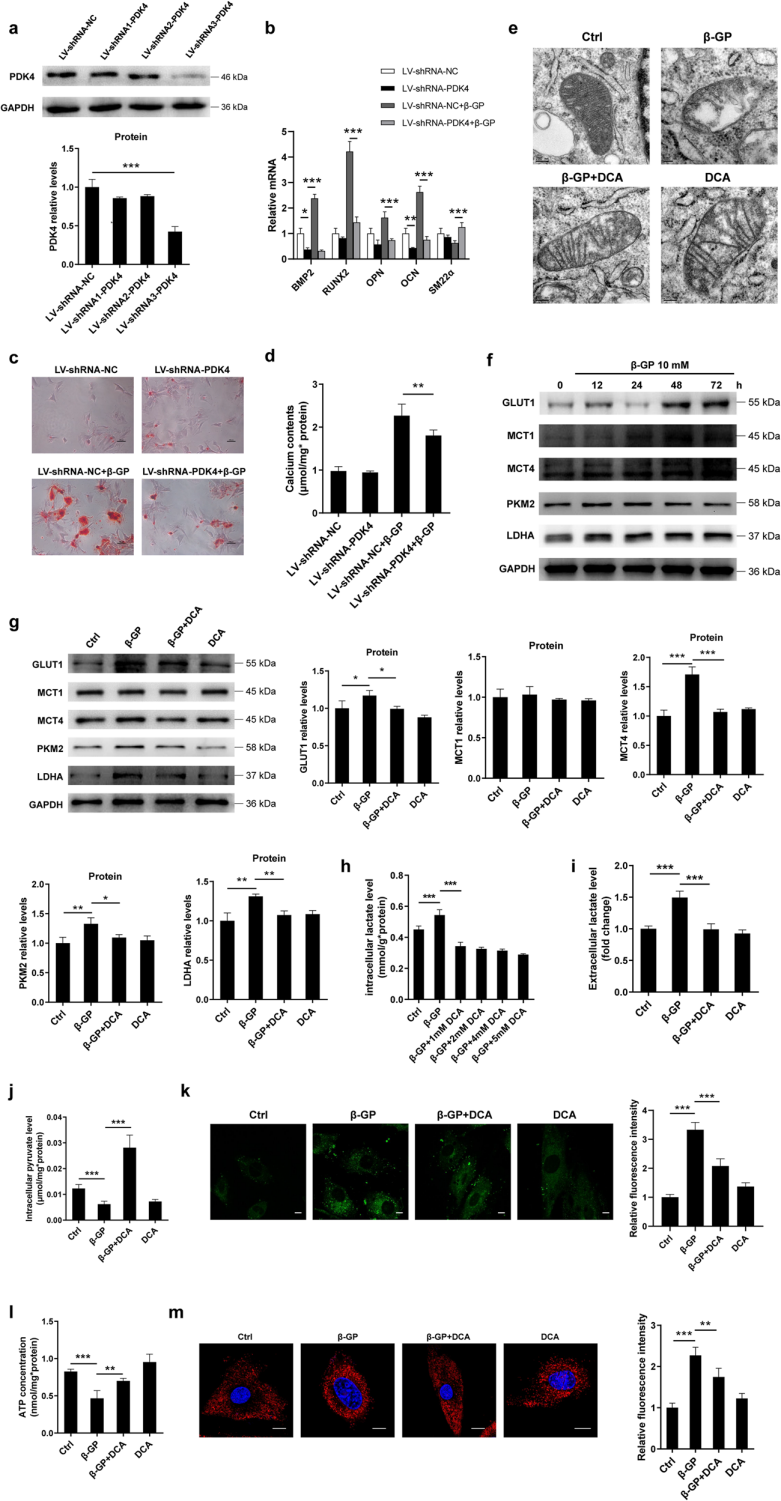
PDK4 promotes the calcification and glycolysis in VSMCs. **a** The transfection efficiency was determined by western blotting. *N* = 3 independent experiments. **b** VSMCs were transfected with lentivirus carrying shRNA targeting PDK4 or NC-shRNA for 48 h and then incubated in the presence or absence of β-GP. The mRNA levels of BMP2, RUNX2, OPN, OCN, and SM22α were measured by RT-qPCR. *N* = 3 independent experiments. **c** Calcium deposition was visualized by Alizarin red S staining at the light microscopic level (original magnification, ×100; Scale bars = 100 μm). *N* = 5 independent experiments. **d** The calcium content was measured by calcium assay kit. *N* = 5 independent experiments. **e** Representative TEM images (scale bar = 100 nm) of the VSMCs treated with 1 mM DCA in the presence or absence of β-GP. **f**–**g** Protein expression of GLUT1, PKM2, LDHA, MCT1 and MCT4 was measured by western blotting. *N* = 3 independent experiments. **h**–**i** Intracellular and extracellular lactate production was measured using a lactate assay kit. *N* = 5 independent experiments. **j** Pyruvate levels were measured. *N* = 5 independent experiments. **k** Glucose uptake was measured using a fluorescent analogue of glucose, 2-NBDG. Scale bars: 10 μm, *N* = 40–50 cells per group. **l** ATP concentration was measured using an ATP determination kit. *N* = 5 independent experiments. **m** Mitochondrial function was evaluated by confocal microscopy using the fluorescent probe MitoSOX. Scale bars: 10 μm, *N* = 40–50 cells per group. *P < 0.05, **P < 0.01, ***P < 0.001.

To investigate the functional role of PDK4 in vascular calcification, VSMCs were transfected with lentivirus-mediated short hairpin RNA (shRNA) targeting PDK4. We tested three different shRNA constructs, of which we selected the one with the strongest knockdown efficiency evident at protein level (Fig. 2a). Several osteoblast markers, including bone morphogenetic protein 2 (BMP2), runt-related transcription factor 2 (RUNX2), osteopontin (OPN) and osteocalcin (OCN), as well as VSMCs contractile marker smooth muscle 22α (SM22α), were assessed by RT-qPCR. Compared with the levels in the cells transfected with NC-shRNA, the β-GP-treated VSMCs with shRNA-mediated PDK4 knockdown showed a decrease in the mRNA levels of BMP2, RUNX2, OPN and OCN, whereas the opposite trend was observed for the mRNA level of SM22α (Fig. 2b). Furthermore, the Alizarin red S staining analysis showed an increase in calcium mineralization in the β-GP-treated VSMCs compared with the mineralization shown in the normal controls, and knocking down PDK4 attenuated this effect (Fig. 2c). Similar result was also obtained by calcium assay kit (Fig. 2d). In addition, we detected the viability and proliferative capacity of cells after knockdown of PDK4 using the Cell Counting Kit-8 (CCK-8) and BrdU assays, respectively. We found that under calcification conditions, knockdown of PDK4 showed an increase in the viability and proliferative capacity of cells compared with NC-shRNA-transfected VSMCs (Supplementary Fig. S1a–b). These results suggest that PDK4 is involved in the control of VSMCs calcification.

We then investigated whether PDK4 affects the mitochondrial network. We first determined the mitochondrial morphology of the VSMCs by transmission electron microscopy (TEM). Compared with their morphology in the normal cells, the mitochondria in the β-GP-treated VSMCs were frequently swollen and displayed altered and perturbed cristae, indicating disrupted mitochondrial integrity in the calcified VSMCs (Fig. 2e). However, DCA treatment attenuated the disruption of the mitochondrial ultrastructure (Fig. 2e). Because of these remarkable changes in mitochondrial structure, we then investigated whether PDK4-mediated mitochondrial structure affects cellular metabolites. We detected the expression of several glycolytic genes, including glucose transporter 1 (GLUT1), pyruvate kinase M2 (PKM2), lactate dehydrogenase A (LDHA), monocarboxylate transporter 1 (MCT1) and MCT4. We observed that the protein expression of the most of glycolytic genes, including GLUT1, PKM2, LDHA and MCT4 was increased upon β-GP treatment in a time-dependent manner, whereas inhibition of PDK4 with DCA partially abolished this effect (Fig. 2f–g). Meanwhile, we compared the levels of glycolysis-related proteins in the PDK4-shRNA and NC-shRNA cells in the presence or absence of β-GP. Under calcification conditions, the cells with shRNA-mediated PDK4 knockdown showed inhibited expression of the GLUT1, PKM2, LDHA and MCT4 proteins compared with NC-shRNA-transfected VSMCs (Supplementary Fig. S2). These observations indicate that PDK4 influences glycolysis-related gene expression.

Then, we sought to determine whether PDK4 modulates the glycolytic phenotype in cultured cells. We observed that both intracellular and extracellular lactate production of VSMCs were significantly increased upon β-GP treatment, and this trend was abolished by DCA (Fig. 2h–i). Furthermore, inhibition of PDK4 upregulated pyruvate levels and reduced glucose uptake, together with increased ATP production, in the VSMCs (Fig. 2j–l). In addition, we evaluated the function of mitochondria with MitoSOX and observed that inhibition of PDK4 attenuated β-GP-mediated mitochondria damage (Fig. 2m).

We also investigated the effect of PDK4 on glucose metabolism in vivo. Several genes involved in the glycolysis pathway were detected by RT-qPCR. The mRNA levels of GLUT1, PKM2, LDHA and MCT4 were significantly increased in the VDN group, while decreased in the DCA + VDN group (Supplementary Fig. S3a). We also detected the protein expression of LDHA and PKM2, which involved in the decarboxylation of phosphoenolpyruvate to pyruvate and generates ATP, and the reduction of pyruvate into lactate, respectively. Western blotting analysis showed that DCA treatment could decrease LDHA and PKM2 expressions compared with the VDN group (Supplementary Fig. S3b). In addition, the serum lactate levels were also elevated in the VDN group compared with the NC group, while DCA treatment inhibited lactate production (Supplementary Fig. S3c). Therefore, these findings suggest that PDK4 promotes VSMCs calcification and mitochondrial fragmentation, accompanied by metabolic reprogramming with high glycolysis levels.

### Autophagy impairment with lysosomal dysfunction is observed under calcification conditions

**Fig. 3:**
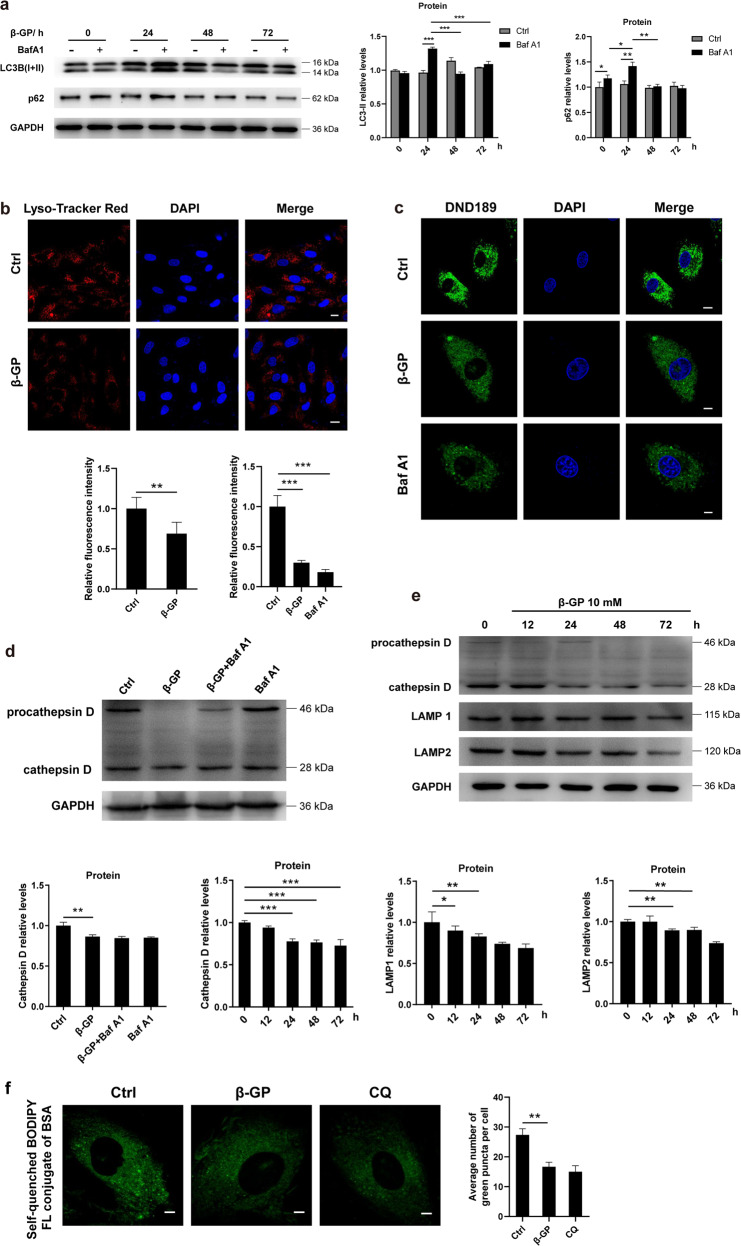
Autophagy is inhibited in the β-GP-treated cells. **a** VSMCs were cultured with 10 mM β-GP in the presence or absence of 80 nM Baf A1 for different periods (0–72 h), and the protein expression levels of LC3 and p62 were measured by western blotting. *N* = 3 independent experiments. **b** The lysosome content of the VSMCs was probed with LysoTracker Red, and images were taken under a confocal microscope. Scale bars = 10 μm, *N* = 40–50 cells per group. **c** The lysosomal pH levels of the VSMCs with or without β-GP treatment were measured by imaging the LysoSensor Green DND-189 probe. Scale bars = 10 μm, *N* = 30–40 cells per group. **d** VSMCs were exposed to 10 mM β-GP in the absence or presence of Baf A1 (80 nM) for 24 h. Protein expression of cathepsin D was determined by western blotting. *N* = 3 independent experiments. **e** VSMCs were treated with 10 mM β-GP for different time periods (0–72 h), and the protein expression levels of cathepsin D, LAMP1 and LAMP2 were measured by western blotting. *N* = 3 independent experiments. **f** Lysosomal degradation was assessed using the self-quenched BODIPY FL conjugate of BSA. Scale bars = 10 μm, *N* = 30–40 cells per group. *P < 0.05, **P < 0.01, ***P < 0.001.

Previous studies revealed that metabolic stress is an activator of autophagy in cardiovascular disease^27^. To identify whether autophagy may be altered during VSMCs calcification, autophagy-related genes were measured by western blotting. Incubation of the VSMCs with β-GP resulted in a decrease in autophagic flux, as measured by LC3-II levels, with the maximum effect observed at 24 h (Fig. 3a). Concomitantly, the protein levels of p62 were elevated at 24 h and then were markedly decreased from 24 to 48 h (Fig. 3a). The treatment of the VSMCs with bafilomycin A1 (Baf A1), an inhibitor of late-stage autophagy, failed to further upregulate the p62 protein levels in the β-GP-treated VSMCs, suggesting that the degradation capacity of the lysosomes was likely low (Fig. 3a).

An acidic lysosomal lumen is essential for the optimal function of resident hydrolases; thus, we analysed lysosomal function under calcification conditions. By tracking lysosomes with LysoTracker Red, a fluorescent dye that labels acidic organelles in live cells, we observed that LysoTracker Red puncta were markedly decreased in the VSMCs exposed to β-GP (Fig. 3b), indicating the possibility that basal lysosome function was suppressed. We then measured lysosomal acidification in the VSMCs using LysoSensor Green DND-189 dye, which emits green fluorescence upon entering acidic lysosomes. In the positive controls, brief treatment with Baf A1 caused a drastic decrease in lysosomal acidification (Fig. 3c), which is a well-established method to decrease acidity within lysosome compartments. The VSMCs showed impaired lysosomal acidification, as revealed by fewer acidic vesicular organelle being formed upon β-GP treatment, suggesting that the concentration of H^+^ in the lysosomes had decreased (Fig. 3c). Given that an acidic lysosomal lumen is essential for the optimal function of lysosomal hydrolases, we detected changes in the proteolytic capacity upon β-GP exposure. Inhibition of autolysosome acidification by either Baf A1 or β-GP decreased the cathepsin D/procathepsin D ratio in the VSMCs (Fig. 3d). Furthermore, the conversion of procathepsin D to cathepsin D was markedly decreased upon β-GP treatment in a time-dependent manner (Fig. 3e). These data suggest that β-GP treatment impairs lysosomal acidification. In addition, we also determined the levels of lysosome-associated membrane proteins 1 (LAMP1) and LAMP2 and observed that the protein levels of LAMP1 and LAMP2 were significantly decreased in a time-dependent manner following β-GP treatment (Fig. 3e). These results consistently suggested a rapid decrease in lysosomal function following β-GP treatment. To strengthen the evidence that β-GP affects autophagic degradation, we evaluated the lysosome degradation capability using the self-quenched BODIPY FL conjugate of BSA. When the self-quenched BODIPY FL conjugate of BSA is degraded under normal lysosomal conditions, it releases green fluorescence in the lysosome. Treatment of the cells with either β-GP or lysosomal inhibitor chloroquine (CQ), caused a significant decrease in the self-quenched BODIPY FL conjugate of BSA-related green fluorescence intensity compared with that of the control cells, which reflects dysfunctional lysosomal degradation (Fig. 3f). These data indicate autophagy impairment with a decrease in lysosomal degradation capacity under calcification conditions.

### PDK4 negatively regulates autophagy concomitant with changes in lysosomal degradation capability, which is correlated with nuclear localization of TFEB

**Fig. 4:**
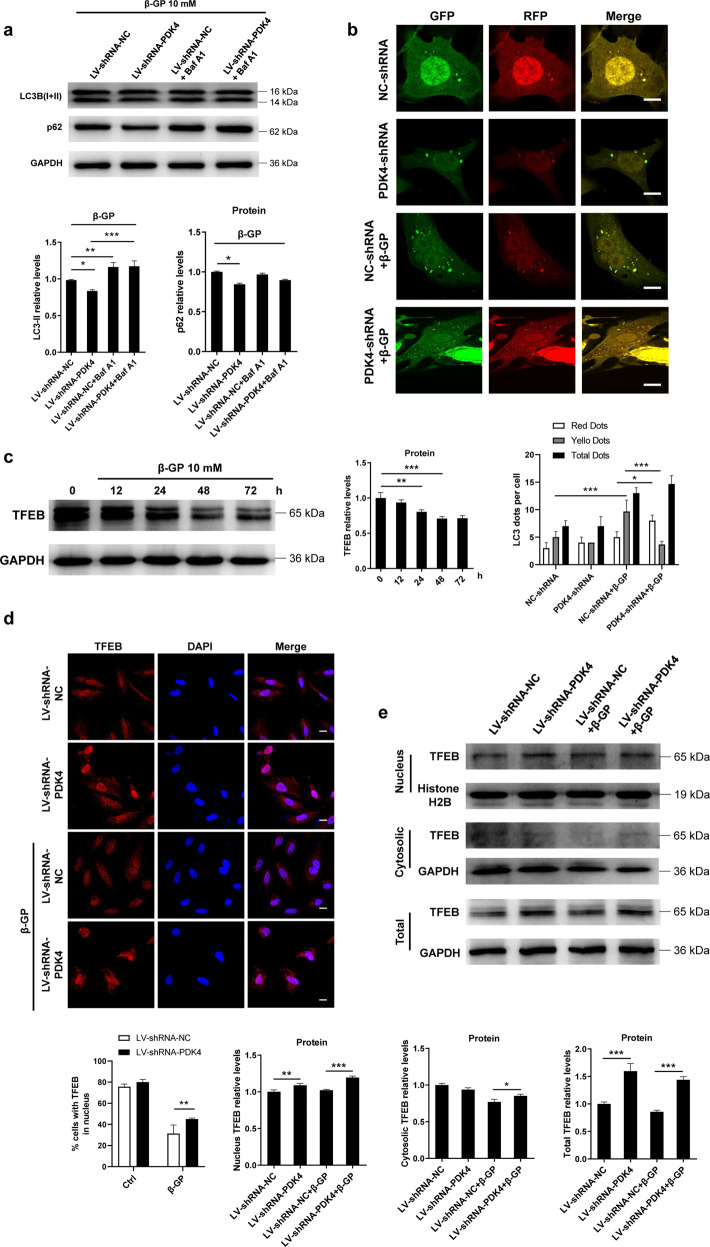

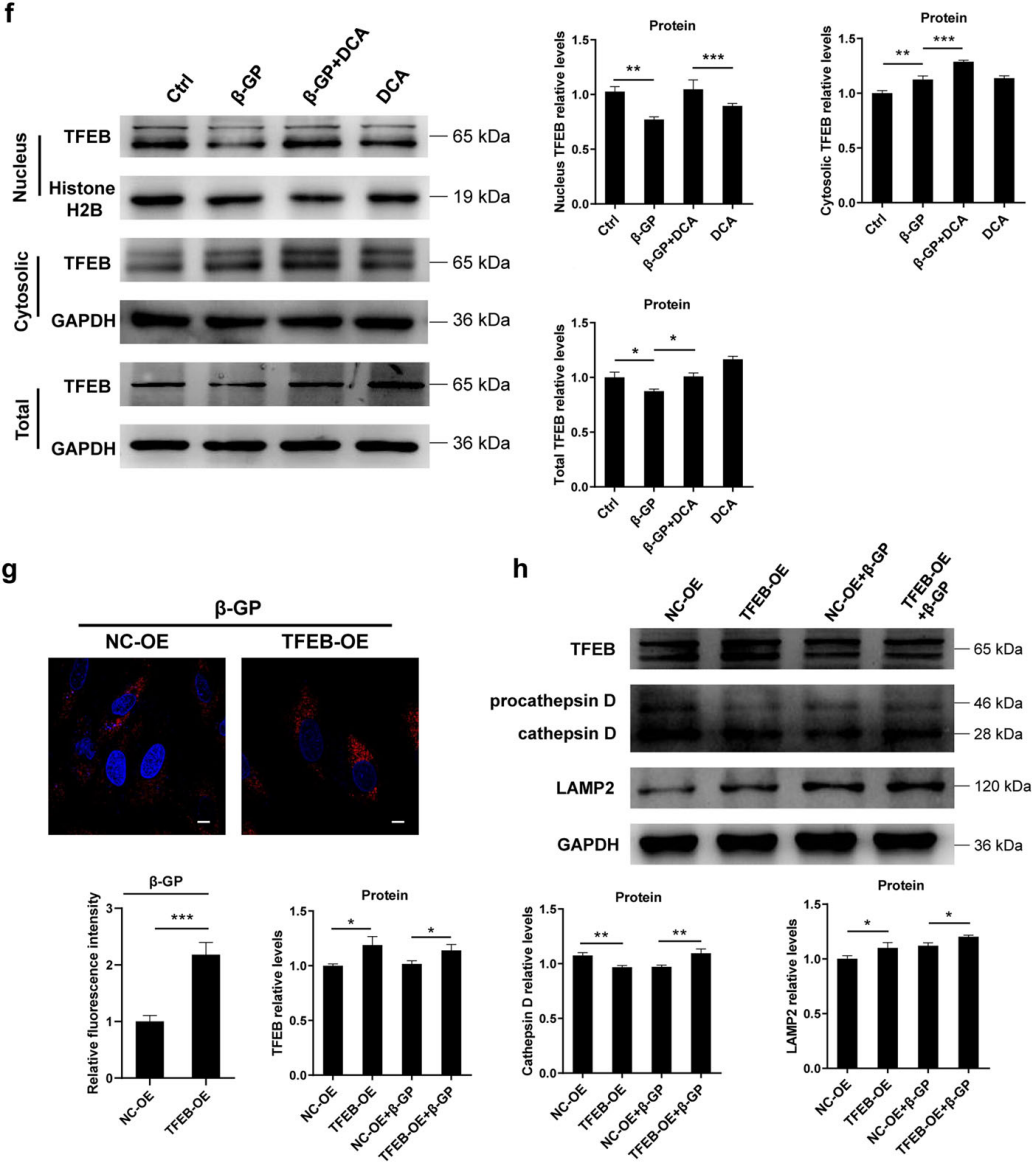
Inhibition of PDK4 promotes autophagy activity. **a** VSMCs were transfected with lentivirus carrying PDK4 shRNA and then exposed to β-GP for 24 h. The protein expression levels of LC3 and p62 were measured by western blotting. *N* = 3 independent experiments. **b** Confocal microscopy of the lentivirus-mRFP-GFP-LC3-transduced VSMCs. Scale bars: 10 μm, *N* = 40–50 cells per group. **c** VSMCs were treated with 10 mM β-GP for 24 h, and TFEB protein expression was determined by western blotting. *N* = 3 independent experiments. **d** VSMCs were transfected with shRNA targeting PDK4 for 48 h. Then, the cells were incubated in the presence or absence of β-GP. After 24 h, the VSMCs were stained with an antibody against TFEB and analysed by confocal microscopy. Scale bars: 10 μm, *N* = 40–50 cells per group. **e** Western blotting analysis of the total, nuclear, and cytoplasmic fractions from PDK4-shRNA-transfected VSMCs in the presence or absence of β-GP. *N* = 3 independent experiments. **f** Cells were treated with 1 mM DCA in the presence or absence of β-GP for 24 h. TFEB protein levels in the total, cytosolic and nuclear fractions were determined by western blotting. *N* = 3 independent experiments. **g** The lysosome content of the VSMCs was probed with LysoTracker Red, and images were taken under a confocal microscope. Scale bars = 10 μm. *N* = 40–50 cells per group. **h** Western blotting analysis of LAMP2, TFEB and Cathepsin D expression in the calcified VSMCs transfected with a plasmid containing the TFEB gene. *N* = 3 independent experiments. *P < 0.05, **P < 0.01, ***P < 0.001.

We investigated the effect of changed PDK4 levels on autophagic signals in the VSMCs. As shown in Fig. 4a, the VSMCs transfected with PDK4 shRNA, followed by β-GP stimulation, showed significantly increased autophagic flux compared with those transfected with NC-shRNA. To further analyse the autophagy defect in calcified VSMCs, we utilized a tandem fluorescence RFP-GFP-LC3 reporter system introduced by lentivirus infection to monitor autophagic flux in the VSMCs. Fluorescence from GFP but not RFP is quenched in acidic LC3B-positive autolysosomes, while yellow puncta indicated nonacidic autophagosomes. We observed that β-GP-treated VSMCs showed an increased retention of nonacidic autophagosomes but very few autolysosomes compared with NC-shRNA-transfected VSMCs (Fig. 4b). More importantly, under calcification conditions, knockdown of PDK4 results in a significant increase in autolysosomes (red puncta) numbers compared with that of the cells transfected with NC-shRNA, suggesting knockdown of PDK4 promotes lysosome-dependence clearance of autophagosomes (Fig. 4b). In addition, we detected the autophagy in vivo. We found that the protein expression levels of LC3-II and p62 were significantly increased in the VDN group compared with that in the NC group, whereas the protein expression levels of LC3-II and p62 were decreased in the DCA + VDN group, suggesting DCA promotes autophagic degradation (Supplementary Fig. S4). Taken together, these data suggest that knockdown of PDK4 improved autophagic flux under calcification conditions.

Given that TFEB has a pivotal role in the regulation of the target genes involved in autophagy and lysosome biogenesis^16^, we first investigated the expression of TFEB in VSMCs calcification and observed that the protein expression of TFEB was dramatically downregulated upon exposure to β-GP in a time-dependent manner (Fig. 4c). To gain further insight into the effects of PDK4 on the nuclear translocation of TFEB, the cells were transfected with either NC- or PDK4 shRNA, followed by 10 mM β-GP stimulation or not. Confocal microscopic analysis showed that the nuclear amount of TFEB decreased in the β-GP-treated VSMCs, while its translocation was partly restored by PDK4 knockdown (Fig. 4d). Furthermore, TFEB was expressed at lower levels, with a greater fraction in the cytosol, in the β-GP-treated VSMCs when compared to its expression in the controls, and knocking down PDK4 resulted in an increase in nuclear TFEB expression, coupled with a dramatic decrease in cytoplasmic TFEB (Fig. 4e). Similar results were also obtained from pharmacological inhibition of PDK4 by DCA (Fig. 4f). Taken together, these findings suggest that silencing PDK4 promotes the nuclear localization of TFEB.

Then, we sought to investigate whether overexpression of TFEB promotes lysosomal function. We observed that TFEB overexpression was able to significantly increase the lysosomal abundance under calcification conditions, as evidenced by LysoTracker Red staining which selectively stains the acidic lysosomal organelles (Fig. 4g). Next, we determined the role of TFEB on different lysosomal markers by western blotting and founded increased protein levels of genes encoding lysosomal membrane proteins such as LAMP2, as well as the lysosomal enzyme cathepsin D (Fig. 4h). Taken together, these data indicate that inhibition of PDK4 promotes the nuclear localization of TFEB, which improves lysosomal function.

### PDK4 disrupts endoplasmic reticulum-mitochondria contact sites, which interferes with mitochondrial respiratory capacity

**Fig. 5:**
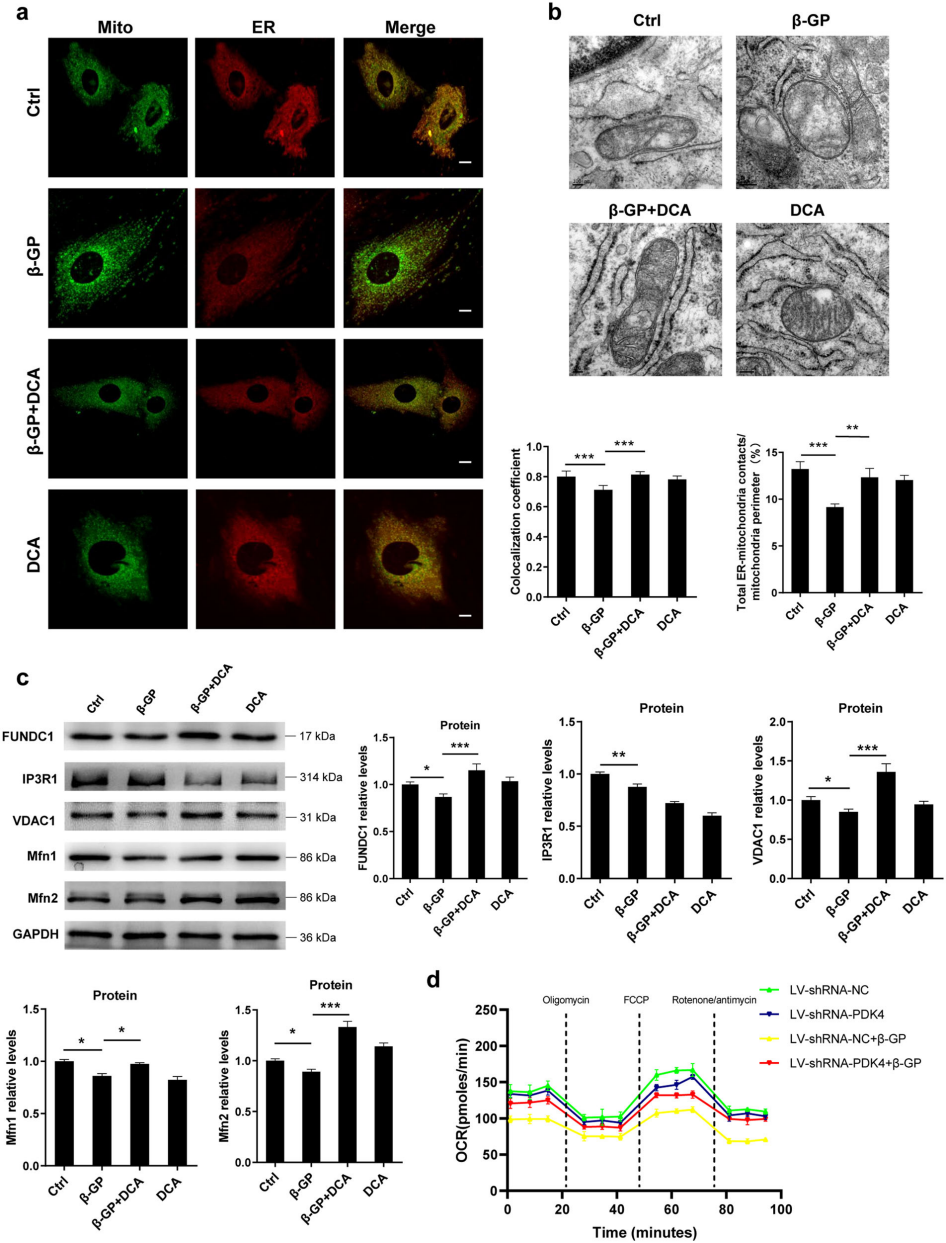
PDK4-mediated mitochondria-associated endoplasmic reticulum membrane disruption correlates with abnormal mitochondrial respiratory capacity. **a** Representative images and quantitative analysis of ER/mitochondria interactions. The ER was stained with ER-Tracker Red, and the mitochondria was stained with MitoTracker Green and then imaged with confocal microscopy. Scale bars = 10 μm. *N* = 40–50 cells per group. **b** Representative TEM images (scale bar = 100 nm) of the VSMCs treated with 1 mM DCA in the presence or absence of β-GP. **c** Protein expression of FUNDC1, IP3R1, VDAC1, Mfn1 and Mfn2 was determined by western blotting. *N* = 3 independent experiments. **d** The OCR was measured by using an Agilent Seahorse extracellular flux analyser. *P < 0.05, **P < 0.01, ***P < 0.001.

Previous studies demonstrated that abnormal intracellular Ca^2+^ homeostasis is involved in high-phosphate VSMCs calcification^28,29^. Given that MAM plays a critical role in Ca^2+^ homeostasis and cellular energetics, we sought to investigate whether abnormal Ca^2+^ homeostasis is mediated by PDK4-related MAM disruption. Using MitoTracker Red and ER-Tracker Green, we determined the extent of ER and mitochondria colocalization. We found a significant reduction in ER-mitochondria contact in the β-GP-treated VSMCs compared to the contact observed in the control cells, and the addition of DCA gradually restored ER-mitochondria contact (Fig. 5a). We also used TEM to investigate the ultrastructural changes of MAM in the VSMCs followed by DCA treatment in the presence or absence of β-GP. We observed that β-GP treatment substantially decreased the ratio of MAM length relative to the mitochondrial perimeter, whereas treatment with DCA alleviated this effect (Fig. 5b). Given that MAM is purportedly composed of a variety of proteins, some of which being inositol 1,4,5-trisphosphate receptor 1 (IP3R1), voltage-dependent anion-selective channel protein 1 (VDAC1), mitofusin 1 (Mfn1), Mfn2 and FUN14 domain-containing 1 (FUNDC1), we evaluated the levels to which these proteins were involved in ER-mitochondria crosstalk under calcification conditions. We found that, for the VSMCs, exposure to β-GP caused a significant decrease in the expression of IP3R1, VDAC1, Mfn1, Mfn2 and FUNDC1 (Fig. 5c). Intriguingly, treatment of the VSMCs with DCA combined with β-GP upregulated the protein expression of VDAC1, Mfn2 and FUNDC1, compared with their expression after β-GP treatment alone, with no significant difference found in the protein level of IP3R1 (Fig. 5c). These data indicate that PDK4 is a negative regulator of ER-mitochondria contact in the VSMCs under calcification conditions.

Given that the integrity of MAM is essential for mitochondrial physiology, we investigated whether the altered MAM caused by PDK4 had a functional impact on mitochondrial energetic activity. Therefore, we detected the mitochondrial respiration activities using Seahorse XF Analyser. The baseline and maximum oxygen consumption rate (OCR) were significantly decreased in the empty vector-transduced cells under calcification conditions, whereas knockdown of PDK4 restored this effect (Fig. 5d). Taken together, these findings suggest that PDK4 impairs mitochondrial respiratory capacity, potentially through the interruption of ER-mitochondria interactions.

### Impairment of mitochondrial energetic function inhibits the lysosomal degradation of autophagic cargo through impaired lysosomal acidification

**Fig. 6:**
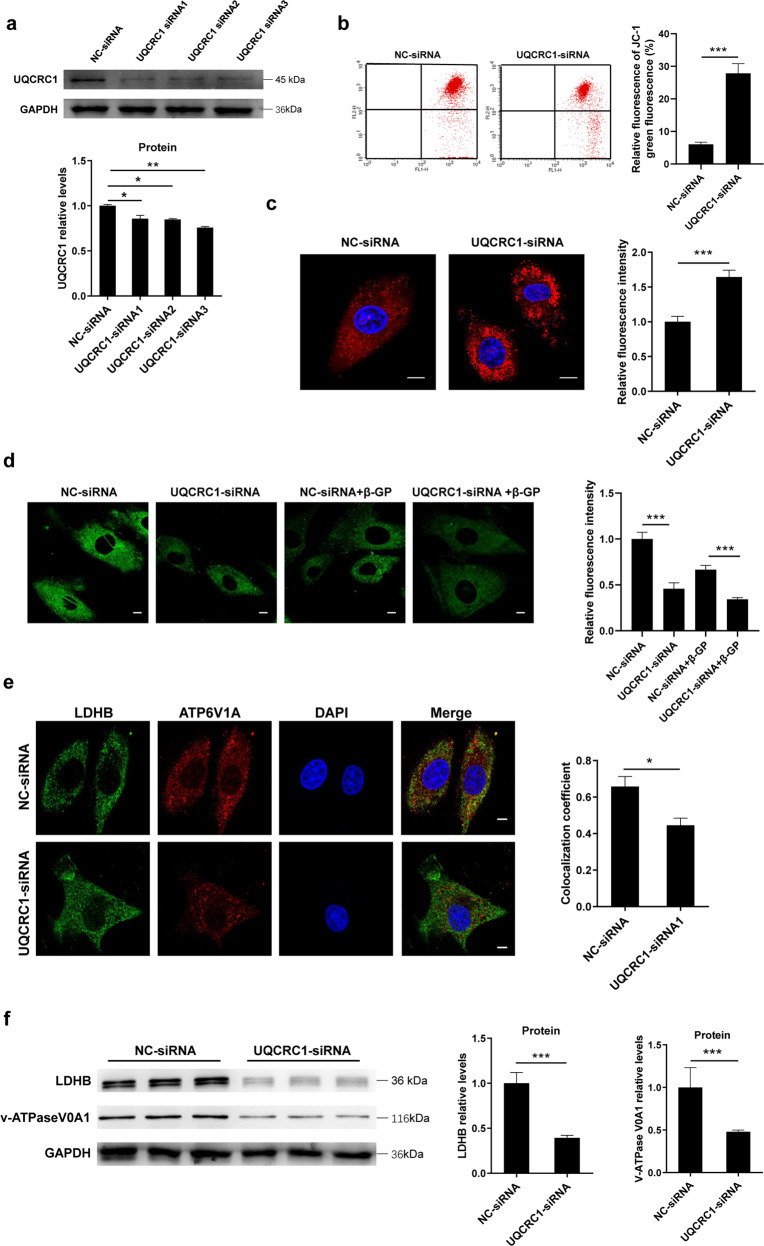
Mitochondrial respiration chain deficiency inhibits lysosomal degradation. **a** VSMCs were transfected with UQCRC1 siRNA or NC-siRNA for 48 h. Then, cells were harvested, and the protein levels of UQCRC1 were analysed by western blotting. *N* = 3 independent experiments. **b** MMP changes were monitored by flow cytometry using JC-1 staining. *N* = 5 independent experiments. **c** Mitochondrial ROS production was evaluated by confocal microscopy using the fluorescent probe MitoSOX. Scale bars = 10 μm. *N* = 40–50 cells per group. **d** Lysosomal degradation was assessed using self-quenched BODIPY FL conjugate of BSA. Scale bars = 10 μm. *N* = 40–50 cells per group. **e** Representative confocal images show LDHB (green channel), v-ATPase V0A1 (red channel), and merged images (yellow channel) puncta in siRNA-UQCRC1-treated VSMCs. Scale bars = 10 μm. *N* = 40–50 cells per group. **f** Protein expression of LDHB and v-ATPase V0A1 was detected by western blotting. *N* = 3 independent experiments. *P < 0.05, **P < 0.01, ***P < 0.001.

Mitochondrial respiratory chain deficiency is a common cause of lysosomal dysfunction^30,31^; therefore, we sought to determine whether changes in lysosomal degradation capacity under calcification conditions, as described above, are a consequence of mitochondrial respiration dysfunction. To confirm this hypothesis, we established a cellular model of chronic mitochondrial respiratory chain deficiency by small interfering RNAs (siRNA)-mediated gene silencing of UQCRC1, a component of complex III in the respiratory chain in the VSMCs (Fig. 6a). Compared with normal VSMCs, siRNA-mediated UQCRC1 knockdown significantly decreased mitochondrial membrane potential (MMP) and increased ROS production in VSMCs, suggesting knockdown of UQCRC1 contributes to mitochondrial dysfunction (Fig. 6b–c). More importantly, cells transfected with siRNA-UQCRC1 showed an evident decrease in the self-quenched BODIPY FL conjugate of BSA-related green fluorescence compared with that in the cells transfected with NC-siRNA or the normal controls, suggesting that the mitochondrial respiratory chain serves as a potential mediator of lysosomal degradation capacity (Fig. 6d).

Next, we further explored the mechanisms underlying the lysosomal inactivation caused by mitochondrial energetic function. Since LDHA favours the reduction of pyruvate to lactate and because lactate dehydrogenase B (LDHB) converts lactate back to pyruvate when interacting with v-ATPase, to yield H^+^, which serves as a major contributor to lysosome acidification^32^, we sought to investigate the protein expression levels and colocalization of LDHB and the v-ATPase V0A1. Intriguingly, the number of LDHB and v-ATPase V0A1 interactions was decreased in the VSMCs transfected with siRNA-UQCRC1 compared to that in the NC-siRNA-transfected cells, indicating that the interaction between LDHB and v-ATPase V0A1 was sensitive to the decrease in mitochondrial respiratory activity (Fig. 6e). Furthermore, the protein expression levels of LDHB and v-ATPase V0A1 were decreased in the siRNA-UQCRC1-transfected cells (Fig. 6f). These results provide evidence that impairment of mitochondrial energetic function inhibits lysosomal acidification.

### Targeting autophagic degradation and glucose metabolism affects VSMCs calcification

**Fig. 7:**
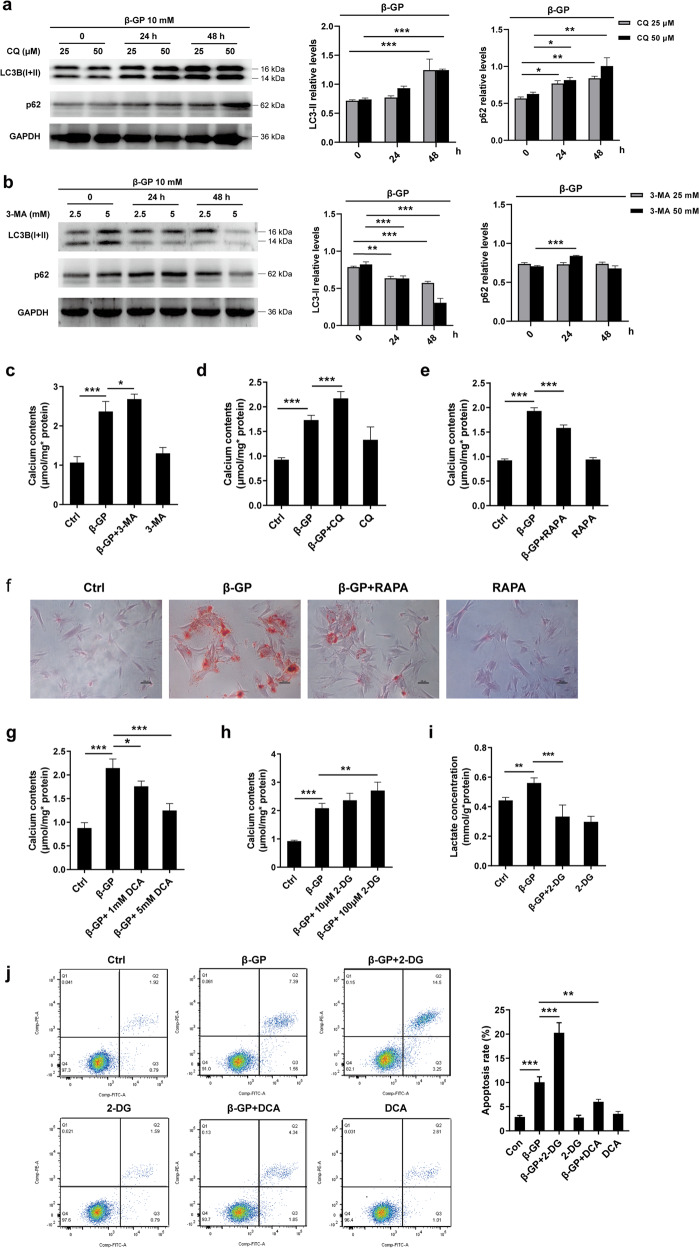

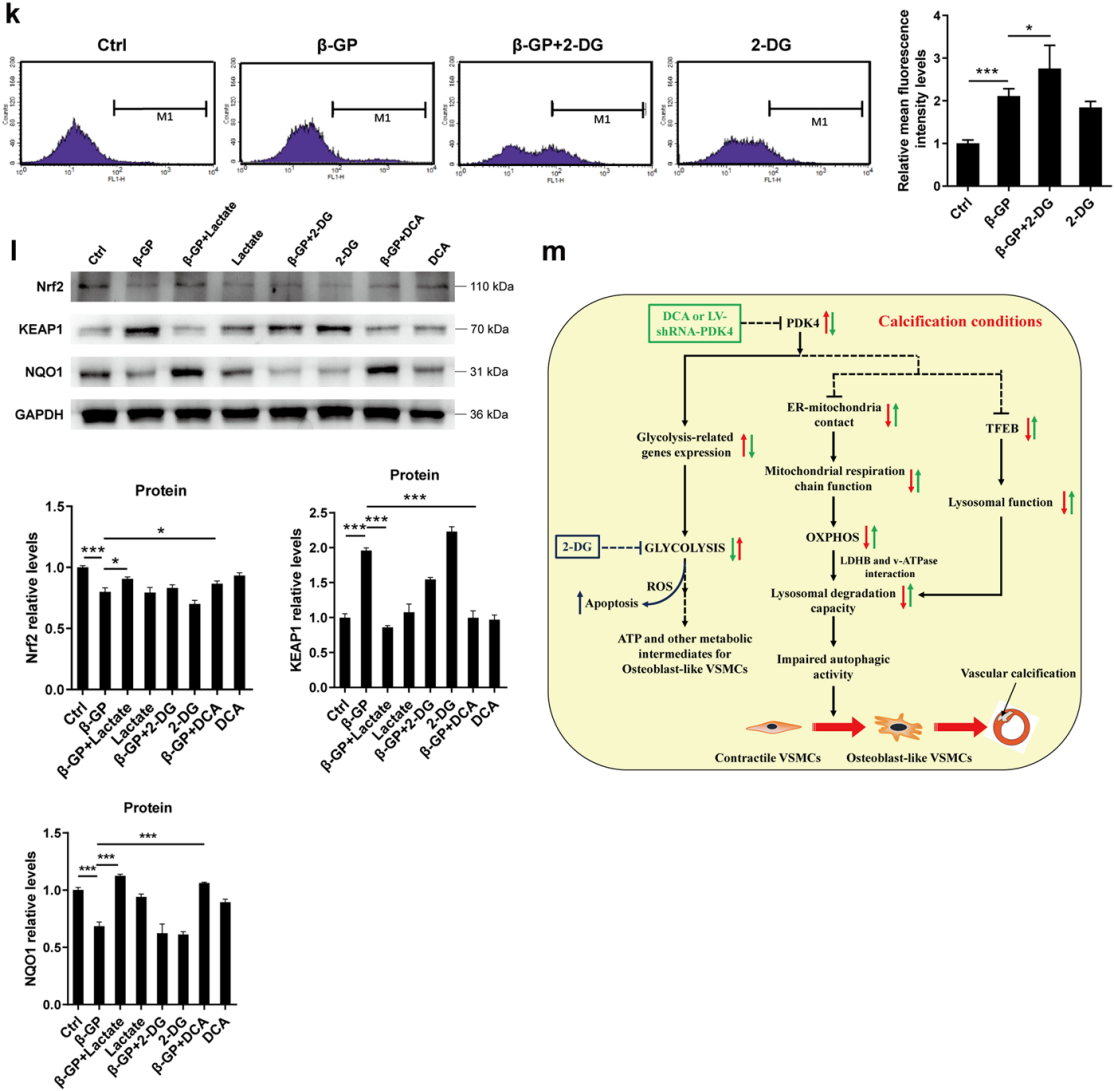
Targeting autophagic-lysosomal function and glucose metabolism affects calcium deposition in VSMCs. **a**, **b** Cells were treated with CQ or 3-MA at the indicated concentrations plus β-GP for 24 h, and the protein expression of LC3-II and p62 was determined by western blotting. *N* = 3 independent experiments. **c**, **d** Cells were incubated with 10 mM β-GP in the absence or presence of CQ or 3-MA for 14 days. Then, the calcium content was measured. *N* = 5 independent experiments. **e**, **f** VSMCs were treated with 10 mM β-GP in the absence or presence of rapamycin for 14 days, and the calcium deposition was visualized by a calcium assay kit and Alizarin red S staining at the light microscopic level (original magnification, ×100; Scale bars = 100 μm). **g** Cells were incubated with 10 mM β-GP in the absence or presence of various DCA concentrations for 14 days, and the degree of calcium deposition was detected by a calcium assay kit. *N* = 5 independent experiments. **h** Cells were exposed to 10 mM β-GP in the absence or presence of various 2-DG concentrations for 14 days, and the calcium deposition was quantitatively analysed using a calcium assay kit. *N* = 5 independent experiments. **i** Lactate production was measured using a lactate assay kit. *N* = 5 independent experiments. **j** Apoptotic cells were measured by flow cytometry with cells stained with Annexin V-FITC in combination with PI. *N* = 3 independent experiments. **k** VSMCs were treated with 10 mM β-GP in the presence or absence of 100 μM 2-DG for 72 h, and ROS production was measured by flow cytometry. *N* = 3 independent experiments. **l** Protein expression of Nrf2, Keap1 and NQO1 was detected by western blotting. *N* = 3 independent experiments. *P < 0.05, **P < 0.01, ***P < 0.001. **m** Proposed mechanisms regarding the effects of PDK4 on vascular calcification: Disrupted ER-mitochondria interactions caused by upregulation of PDK4 contributes to impaired mitochondrial respiration, which decreases lysosomal function via inhibition of LDHB and v-ATPase A1 interaction. Meanwhile, PDK4 inhibits lysosomal function via inhibition of the nuclear translocation of TFEB. These consequences collaborate and contribute to impaired autophagic activity, which has been implicated in the pathogenesis of vascular calcification. In addition, PDK4 drives metabolic reprogramming of VSMCs, with a higher rate of glycolysis under calcification conditions; while inhibition of glycolysis promotes apoptosis in VSMCs.

We first investigated whether there is a causal relationship between decreased autophagic flux and the progression of VSMCs calcification. VSMCs were treated with either CQ or 3-MA, an early-stage autophagy inhibitor. As expected, CQ and 3-MA, each blocked autophagic flux in the VSMCs (Fig. 7a–b). Furthermore, there was an increase in calcium deposition in both the CQ- and the 3-MA-treated VSMCs (Fig. 7c–d). These findings indicate that interrupted autophagic flux aggravated VSMCs calcification.

Then, we sought to investigate whether upregulation of autophagy could reduce calcium deposition in the VSMCs. Most of the autophagic signal was proven to be mammalian target of rapamycin (mTOR)-dependent. Furthermore, it has been reported that mTORC1 activity could affect lysosomal function^33^. For these reasons, we investigated whether β-GP alters mTOR signalling in VSMCs. The results of western blotting analyses showed that β-GP elevated p-mTOR and its downstream target protein p-S6K levels in a time-dependent manner, suggesting mTOR signalling is activated in VSMCs calcification (Supplementary Fig. S5a). Elevated p-S6K levels in β-GP-treated VSMCs was suppressed by rapamycin (RAPA), an mTOR-dependent autophagy inducer (Supplementary Fig. S5b). More importantly, incubation of the cells with rapamycin alleviated β-GP-induced calcium deposition in the VSMCs, as evidenced by quantitative determination of calcium and Alizarin Red staining (Fig. 7e–f). In addition, we also investigated the effects of 3-MA or RAPA on the cell viability and proliferative capacity of VSMCs. We found that supplementation of VSMCs with 3-MA resulted in an increase in proliferation capacity of VSMCs compared with that of the untreated cells, despite the cell viability did not change significantly (Supplementary Fig. S6a and S6c). In contrast, incubation of the cells with RAPA reduced the cell viability and the proliferation capacity of VSMCs (Supplementary Fig. S6b and S6d). These findings indicate that impaired autophagic flux is critically involved in β-GP-induced VSMCs calcification.

Given that PDK4 promotes a Warburg effect in calcified VSMCs, as described above, we investigated whether alternation of glucose metabolism could affect VSMCs calcification. Facilitating the oxidation of glucose via the stimulation of PDH by DCA showed a decrease in calcium deposition in the β-GP-treated VSMCs compared with that in control cells treated with β-GP alone (Fig. 7g). Intriguingly, inhibition of glycolysis with 2-deoxy-D-glucose (2-DG) suppressed β-GP-induced lactate production but additionally accelerated calcium deposition in the VSMCs (Fig. 7h–i). Furthermore, supplementation of VSMCs with 2-DG resulted in a decrease in autophagic flux of VSMCs compared with that of the untreated cells, as evidenced by the decreased LC3-II and p62 expression (Supplementary Fig. S7). These findings suggest that enhanced glycolysis has a protective effect against VSMCs calcification.

Next, we sought to investigate the molecular mechanisms involved in it. Incubation of the cells with 2-DG and β-GP together induces VSMCs apoptosis compared with the effect of β-GP treatment alone, whereas DCA treatment enhanced cell survival during a challenge by β-GP-induced apoptosis, as demonstrated by a marked decrease in the number of apoptotic cells (Fig. 7j). Noteworthy, incubation of the cells with 2-DG did not induce VSMCs necrosis compared with that of the control cells (Fig. 7j). Lactate dehydrogenase (LDH) is a cytosolic enzyme used as a tool to test cell membrane integrity and necrosis, which could be released from damaged cell membranes. The LDH activity did not change markedly in both 2-DG-treated VSMCs and control VSMCs (Supplementary Fig. S8). We also observed that inhibition of glycolysis exacerbated β-GP-induced ROS production compared to the levels induced by β-GP treatment alone (Fig. 7k). These findings suggest that enhanced glycolysis is essential for VSMCs to escape apoptosis and oxidative stress damage.

Damage to the vascular microenvironment by either oxidative stress or apoptosis plays an important role in the pathogenesis of vascular calcification. Considering that glycolysis is beneficial for cells combating a highly oxidative microenvironment to attain better survival and that its intermediates are required for the growth and survival of rapidly dividing cells^34^, we hypothesized that lactate, a calcification microenvironment factor, may enable VSMCs to develop oxidative stress resistance to β-GP-induced apoptosis. Since NF-E2-related factor 2 (Nrf2)/Kelch-like ECH-associated protein 1 (Keap1) signalling is one of the main intracellular antioxidant pathways by which cells resist oxidative stress damage, we hypothesized that lactate enhances oxidative stress resistance against apoptosis through Nrf2 signalling. The protein expression of Nrf2 and its downstream gene NQO1 was significantly increased in response to lactate in the presence of β-GP compared with that of β-GP treatment alone (Fig. 7l), suggesting that lactate promoted the dissociation of Nrf2/Keap1 and activated the Nrf2-mediated antioxidant cascade in the VSMCs. A similar effect was observed upon DCA treatment (Fig. 7l). However, 2-DG treatment inhibited the expression of Nrf2 (Fig. 7l). Meanwhile, previous studies identified that the upregulation of Nrf2 could enhance the transcription of cytoprotective genes including p62, which in turn increases Nrf2 activity by inactivating Keap1 to produce a positive feedback loop. Therefore, we detected the expression of p62. We observed that compared with the β-GP-treated VSMCs, supplementation with either lactate or DCA promotes the protein expression of p62, suggesting the non-canonical activation of the Nrf2/Keap1 system (Supplementary Fig. S9). Taken together, these findings indicate that, under calcification conditions, glycolysis serves as a metabolic adaptation to ensure the survival of differentiated VSMCs via lactate-mediated activation of p62/Nrf2/Keap1 signalling.

## Discussion

In the present study, we identified a novel and unexpected role of PDK4 in vascular calcification and attained several interesting findings. First, PDK4 expression is increased in calcified aorta tissues of rats and upon calcification of VSMCs. Either genetic or pharmacological inhibition of PDK4 attenuates β-GP-induced calcium deposition in VSMCs. Second, PDK4-mediated MAM disruption contributes to the decrease in mitochondrial energetic function. We also established a cellular model of chronic mitochondrial respiratory chain deficiency and found that impaired mitochondrial respiratory capacity inhibits lysosomal function by interfering with V-ATPase and LDHB interactions. Third, PDK4 inhibits TFEB nuclear translocation, which is associated with a decrease in lysosomal function. Fourth, PDK4 facilitates glycolytic metabolism, as characterized by increased expression of key glycolytic enzymes and higher rates of glucose uptake, and apparently contributes to defective mitochondrial respiration. In addition, glycolysis serves as a metabolic adaptation to improve VSMCs oxidative stress resistance. These findings provide novel evidence for the supposition that PDK4 is a possible therapeutic target for anti-calcification intervention.

Using a series of functional measurements, particularly concerning the evaluation of lysosomal degradation, we found that the overall autophagic activity was decreased during the calcification process. Furthermore, by using LysoSensor Green DND-189 dye, we also discovered that the degradation capacity of the lysosomes was probably at a low level because of impaired lysosomal acidification. Previous evidence suggested that the MAM is the site for selective autophagosome formation and that the mitophagy receptor FUNDC1 interacts with LC3 to recruit autophagosomes^35^. In the current study, FUNDC1 expression was decreased in the calcification microenvironment, concomitantly with disrupted ER-mitochondrial contact sites, and therefore, defective mitochondria could not be selectively engulfed by autophagosomes. Notably, we found that PDK4 inhibition promoted the expression of FUNDC1, accompanied by enhanced autophagic flux. This observation is consistent with previous observations that also reported that DCA could induce autophagy, as confirmed by the formation of autophagosomes^36^. In addition, we addressed the effects of autophagy modulators on calcium deposition in VSMCs. This result showed that either 3-MA or CQ can accelerate calcium deposition in VSMCs triggered by β-GP. In addition, since most of the autophagic signal is mTOR-dependent, we stimulated autophagy activity via mTOR suppression by rapamycin and observed a significant decrease in calcium deposition in the VSMCs. On the other hand, autophagic flux restoration was important for VSMCs against calcium deposition.

High glycolysis rates and low glucose oxidation rates were observed during the calcification process. This change is similar to Warburg effect which is observed in most cancer cells characterized by the increased metabolism of glycolysis even in the presence of oxygen, resulting in increased lactate production and reduced mitochondrial oxidation of pyruvate^37^. These findings are also consistent with previous observations that reported by Leem et al.^38^. In addition to producing ATP, this metabolic shift could provide metabolic intermediates needed for VSMCs phenotype switching. However, this change might cause an increased uncoupling of glycolysis from glucose oxidation, resulting in the production of protons, which would result in a series of events that could alter ionic homeostasis and result in ATP being diverted from the maintenance of cell function and towards re-establishing ionic homeostasis, thereby decreasing energy efficiency. Simultaneously, we found that, under calcification conditions, lactate counteracted the oxidative stress induced by β-GP, as evidenced by Nrf2 signalling activation, although the reaction intermediates have been traditionally viewed as a metabolic junk. Intriguingly, the inhibition of glycolysis by either DCA or 2-DG produced two opposite effects on VSMCs calcification. The possible reasons for this discrepancy under calcification conditions may be explained by glycolysis serving as a metabolic adaptation to ensure the survival of differentiated VSMCs and by the suppression of the ‘Warburg effect’ by DCA contributing to a strong reactivation of oxidative phosphorylation and increased redox status, as assessed by the constitutive expression of Nrf2.

The proper function of mitochondria and ER is of importance to cellular homeostasis, whereas dysfunction at either site or perturbation of ER-mitochondria contact has also been reported in various models of metabolic-related diseases, including insulin resistance^39^, diabetic cardiomyopathy^40^ and obesity^24^. Mitochondrial dysfunction contributes to accelerated cell apoptosis, inflammation, excessive ROS production, lactate accumulation, and a deficient ATP supply due to the reduced oxidative phosphorylation of glucose, and all of these factors could promote vascular calcification^41,42^. Similarly, ER stress-mediated apoptosis is also involved in vascular calcification^43^. However, the mechanistic role of ER-mitochondria contact in vascular calcification remains unclear. Our data, for the first time, show that the disruption of ER-mitochondria interactions caused by the upregulation of PDK4 impairs mitochondrial energetic metabolism in VSMCs. Intriguingly, these structural and functional alterations of MAM were partially restored by DCA treatment, as evidenced by the TEM evidence and the colocalization of the ER and mitochondria. These outcomes provide a strong link between PDK4-mediated abnormal MAM and energy metabolic insufficiency in calcified VSMCs. Under such specific conditions, decreased transcription and inhibited activity of the mitochondrial respiratory complex may result in a failure to capture free electrons, evoking ROS bursts and triggering subsequent mitochondrial damage^44^. Furthermore, ROS generation induced by other cellular sources or accumulated metabolites can also contribute to a secondary impairment of respiratory chain components with a further increase in superoxide production by mitochondria^45^. In addition, several studies suggested that alteration of ER-mitochondria crosstalk may lead to a disruption of inter-organelle Ca^2+^ transfer and subsequent ER stress^46^, thus further studies are required to clarify the role of inter-organelle Ca^2+^ exchange in regulating vascular calcification.

Recent evidence suggests that FUNDC1 is an integral component of the ER-mitochondria microdomain, which modulates Ca^2+^ homeostasis, mitochondrial fission, and mitophagy^47^. Thus, we further investigated whether PDK4-driven ER-mitochondria contact disruption is associated with FUNDC1 downregulation. We observed that the protein levels of VDAC1, Mfn1, Mfn2 and FUNDC1, which are involved in the regulation of MAM formation and function, were significantly increased upon DCA treatment under calcification conditions, whereas no change in the levels of IP3R1 was observed. Csordas et al.^48^ reported that the absence of IP3Rs does not affect the ER-mitochondrial connection. Intriguingly, several studies have shown the positive effect of PDK4 on ER-mitochondria interactions in an obesity-induced insulin resistance model^24^. These apparent discrepancies might be due to different models and the distinct metabolic characteristics of the cells that were studied. Furthermore, ER-mitochondria coupling is highly dynamic and requires appropriate membrane organization, and its regulation is a complex process involving multiple genes. Notably, accumulating evidence has suggested the involvement of MAM in autophagy regulation, referring to autophagosome assembly and maturation^26,49,50^, indirectly confirming that the blockage of autophagic flux caused by PDK4 is related to the disruption of MAM. These observations highlight the complexity of the subcellular compartment and function in MAM, as either increasing or decreasing ER-mitochondria interactions is likely sufficient to be deleterious.

The role of lysosomes in cellular biology has been expanded in recent years to extend understanding beyond the conventional view that they are cellular trash cans; notably, a recent discovery revealed lysosome involvement in autophagic flux regulation^14,51^. However, the mechanisms that mediate the cross talk between mitochondria and lysosomes have not yet been fully clarified, regardless of whether they transpire through their contact sites or by signalling pathways. Existing evidence suggests that loss of mitochondrial function is tightly related to mitochondrial fragmentation, increased ROS production, and decreased ATP synthesis, each with a potential effect on lysosomes^52^. Here, we show that PDK4-mediated mitochondrial respiratory chain deficiency inhibited lysosomal degradation, which was the most surprising and important observation of this study. We prepared a cellular model of chronic respiratory chain deficiency and found that, under calcification conditions, mitochondrial respiratory chain dysfunction inhibited lysosomal degradation capability, which indicated that maintenance of mitochondrial energetic function is essential for lysosomal activity. These findings are partially in line with previous observations in HeLa cells revealing that chronic mitochondrial respiratory chain deficiency inhibits lysosomal degradation^30^. In addition, Demers-Lamarche et al.^52^ also reported that the structure and function of lysosomes could be caused by defective mitochondrial function. Altogether, these results highlight that mitochondrial respiratory chain deficiency may serve as a targeted molecular site in autophagic flux regulation and that impairment of lysosomal degradation capability might be the underlying mechanism.

In another important finding of this study, we have, for the first time, uncovered a novel pathway by which PDK4 blocks the nuclear translocation of TFEB. Notably, several studies reported that elevated ROS levels could trigger TFEB nuclear translocation in other cell models, despite oxidative stress being accompanied by the entire pathophysiological process of vascular calcification. This discrepancy probably results from the variation in the degree of ROS and dissimilar sensitivity of different cell lines to oxidative stress. Under physiological conditions, a mild increase in ROS levels may act as a “survival” signal, which could stimulate the nuclear translocation of TFEB, facilitating the clearance of damaged mitochondria and restoration of redox homeostasis; however, calcification induction is a relatively long-term process, and the accumulation of ROS may produce severe oxidative damage and serve as a “death” signal, ultimately resulting in inactive or compromised TFEB-related pathways. In addition, recent the evidence suggests that overexpression of TFEB coordinates metabolic flexibility, which controls glucose homeostasis, induces mitochondrial biogenesis, enhances respiratory chain complex activities, and elevates ATP production^53^.

In summary, data from this study highlight a critical role of PDK4 in metabolic reprogramming and autophagy regulation during vascular calcification. These findings increase our understanding of the interaction and cross talk of MAM and lysosomes undergoing degradation and changes in the metabolic microenvironment in terms of vascular calcification. MAM disruption and abnormal TFEB signalling are novel events associated with the mechanisms of action of vascular calcification, finding that may provide new perspectives for the development of future therapies in the field of vascular calcification.

## Materials and methods

### Antibodies and reagents

The antibodies used in our experiments were: anti-FUNDC1 antibody (Sigma, ABC506), anti-LC3 antibody (Proteintech, 14600-1-AP), anti-p62 antibody (Proteintech, 18420-1-AP), anti-LAMP1 (Boster, M00780-1), anti-LAMP2 (Proteintech, 27823-1-AP), anti-PDK4 antibody (Boster, BA3144), anti-cathepsin-D antibody (Boster, PB0020), anti-TFEB antibody (Proteintech, 13372-1-AP), anti-UQCRC1 antibody (Proteintech, 21705-1-AP), anti-Nrf2 antibody (Proteintech, 16396-1-AP), anti-Keap1 antibody (Proteintech, 10503-2-AP), anti-IP3R1 antibody (Boster, PB0223), anti-VDAC1 antibody (Proteintech, 10866-1-AP), anti-Mfn1 antibody (Proteintech, 13798-1-AP), anti-Mfn2 antibody (Proteintech, 12186-1-AP), anti-ATP6V01 antibody (Proteintech, 13828-1-AP), anti-LDHB antibody (Proteintech, 66425-1-Ig). Secondary antibody for western blotting: HRP-conjugated Affinipure Goat Anti-Mouse IgG(H + L) (Proteintech, SA00001-1), HRP-conjugated Affinipure Goat Anti-Rabbit IgG (H + L) (Proteintech, SA00001-2). Secondary antibody for immunofluorescence: CoraLite488-conjugated Affinipure Goat Anti-Mouse IgG (H + L) (Proteintech, SA00013-1), CoraLite594-conjugated Goat Anti-Rabbit IgG (H + L) (Proteintech, SA00013-4).

Reagents: DCA (Selleckchem, S8615, USA), 3-MA (Selleckchem, S2767, USA), Baf A1 (Selleckchem, S1413, USA), CQ (Selleckchem, S4430, USA), Lactate (Solarbio, L8630, China), 2-DG (GlpBio, GC17430, USA).

### Animal models

All animal care and experimental protocols complied with the Animal Management Rule of the Ministry of Health, People’s Republic of China and the Care and Use of Laboratory Animals published by the United States National Institutes of Health and approved by the Animal Care Committee of Southeast University. Male SD rats (180–200 g) were randomly divided into four groups as follows: NC group, VDN group, VDN + DCA group, and DCA group. Rats were housed under standard conditions (room temperature 20 ± 8 °C, humidity 60 ± 10%, lights from 6:00 to 18:00) and given standard rodent chow and free water. To establish vascular calcification model, rats were injected with vitamin D_3_ (300,000 IU/kg) simultaneously intragastric administrated with nicotine (25 mg/kg in 3 mL peanut oil) at 9 a.m. on the first day. The nicotine administration was repeated at 5 p.m. on the same day, and the modelling continued for 8 weeks. Rats in control group received the injection of saline and oral gavage of peanut oil. Rats in DCA + VDN group was intragastric administrated with 50 mg/kg of DCA each day simultaneously induced aortic calcification. Rats in DCA group was intragastric administrated with 50 mg/kg of DCA each day. After 8 weeks of treatment, all rats were euthanized via intraperitoneal injection of pentobarbital followed by exsanguination through cardiac puncture.

### Cell culture and treatments

Primary VSMCs were isolated from the thoracic aortas of 5-week-old male SD rats. Briefly, after medial tissues isolating from segments of rat aortas, they were cut into 1–2-mm^2^ sections and placed in a culture flask with Dulbecco’s modified Eagle’s medium (DMEM, Hyclone) supplemented with 15% foetal bovine serum (FBS, GIBCO). Cells that migrated from the explants were cultured in DMEM supplemented with 15% FBS and 1% penicillin-streptomycin (Hyclone) at 37 °C in a humidified atmosphere with 5% CO_2_. To induce calcification in vitro, growing cells at ~80% confluence were exposed to 10 mM β-GP.

### Alizarin Red S and von Kossa staining

Alizarin Red S staining was performed to determine the calcium deposition in VSMCs. Cells were fixed in 4% polyoxymethylene for 20 min. Then, cells were rinsed twice with phosphate buffered saline (PBS) and stained with 1% alizarin red solution for 10 min at room temperature. Photographs were taken under the light microscope. To quantify Alizarin Red staining, we added 100 μl of hexadecylpyridinium chloride solution (100 mM) to each well and changes in optical density were measured using a spectrophotometer at 560 nm. For von Kossa staining, the sections of isolated aorta tissues were dewaxed and hydrated. Subsequently, samples were incubated with 1% silver nitrate solution for 30 min under an intense sunbeam or ultraviolet light. Samples were then washed and treated with 5% sodium thiosulfate. After several washes, calcified nodules were stained brown to black under the light microscope.

### Transmission electron microscopy

VSMCs were fixed with 2.5% glutaraldehyde, washed twice with PBS, stained with 1% osmium tetroxide, dehydrated with standard ethanol series, and embedded in epoxy resin. Subsequently, the samples were loaded into capsules and polymerized at 60 °C for 24 h. Thin sections were cut using the RMC MT-X ultramicrotome and collected on copper grids. Photographs were collected through the electron microscope (H-7100; Hitachi, Tokyo, Japan). Image J software was used to delimitate the distance between mitochondria and ER. The fraction of mitochondrial membrane in contact with ER within a 50 nm range was calculated and normalized to mitochondria perimeter, and expressed as total percent of contact between mitochondria and ER.

### VSMCs viability and proliferation assays

VSMCs viability was assessed using the CCK-8 cell viability assay. Following the manufacturer’s instructions, VSMCs were seeded into 96-well cell culture plates (5000 cells/well) and incubated overnight to allow attachment. After the indicated treatments, cells were incubated with the 20 μL of CCK-8 solution at 37 °C for 2 h, and the absorbance at 450 nm was measured using a microplate reader. For cell proliferation analysis, a BrdU incorporation assay kit (Roche, USA) was applied to assess VSMCs proliferation according to the manufacturer’s instructions.

### ATP content determination

The ATP levels were measured using an ATP determination kit (Beyotime Institute of Biotechnology, Jiangsu, China) according to the manufacturer’s instruction. Briefly, after the indicated treatments in 6-well plates, samples were lysed in RIPA lysis buffer on ice for 30 min and centrifuged at 12,000 × *g* for 10 min at 4 °C, and cell supernatants were collected. Aliquot of ATP detection working solution was added to a black 96-well culture plate and was incubated for 5 min at room temperature. Then, cell supernatants were added to the wells. The bioluminescence was measured and normalized to protein concentration.

### Measurement of mitochondrial membrane potential by flow cytometry

MMP was determined using the JC-1 membrane potential assay (Beyotime Institute of Biotechnology, Jiangsu, China) according to the manufacturer’s instruction. Briefly, cells were incubated with fresh culture medium containing the fluorescent dye JC-1 for 20 min at 37 °C in the dark. All samples were then washed twice in PBS, resuspended in JC-1 assay buffer, and analysed immediately by flow cytometry at 490 nm excitation. Data were collected at 530 nm emission for green fluorescence of the JC-1 monomer and at 590 nm for red fluorescence of JC-1 aggregates on the filter 2 (FL2 detector). Results were calculated as the red/green fluorescent intensity ratio.

### Western blotting

Total protein from treated cells was extracted with RIPA buffer and quantified by using the BCA Protein Assay Kit (Beyotime Institute of Biotechnology, Jiangsu, China). Proteins (20 µg/lane) were separated by sodium dodecyl sulfate polyacrylamide gel electrophoresis (SDS-PAGE) and transferred onto polyvinylidene fluoride (PVDF) membranes. After blocking with 5% milk in Tris-buffered saline and Tween 20 (TBST, pH 7.6), membranes were incubated overnight at 4 °C with primary antibody. After incubation with second antibodies for 1 h at room temperature, bound proteins were visualized by using ECL (Pierce) and visualized on Tanon-5200 Chemiluminescent Imaging System (Tanon Science & Technology Co., Ltd., Shanghai, China). The relative protein levels were calculated by normalizing to GAPDH protein as a loading reference by using Image J.

### Plasmid transfection, gene silencing and lentiviral infection

TFEB plasmid was purchased from Genechem Biotechnology, Inc. (Shanghai, China). Plasmid DNA was transfected using Lipofectamine 2000 (Life Technologies) according to the manufacturer’s protocol. Three independent sets of siRNAs against UQCRC1 were synthesized by KeyGen Biotechnology, Inc. (Jiangsu, China). The sequences of the UQCRC1 siRNAs were as following: UQCRC1-siRNA1: 5′-GCGCACAGACUUGACUGACUATT-3′; UQCRC1-siRNA2: 5′-GAAGACGCUGUACCUAGUAUATT-3′; UQCRC1-siRNA3: 5′-GGCUUCCGUUGCUGUAGCUAATT-3′. VSMCs transfected with the indicated siRNAs after they reached 70% confluency by using Lipofectamine 2000 according to the manufacturer’s protocol. The proficiency of siRNA transfection was assessed by western blotting. Three independent sets of shRNAs against PDK4 were synthesized by the GenePharma Biotechnology, Inc. (Shanghai, China). The shRNAs sequence targeting PDK4 were as following: PDK4-shRNA1: 5′-GCAGTAGTCGAAGATGCCTTT-3′; PDK4-shRNA2: 5′-GGATTACTGACCGCCTCTTTA-3′; PDK4-shRNA3: 5′-GGAGATCTGAATCTCTATTCC-3′. Rat PDK4-shRNA lentiviral particles were also designed by this company.

### Immunofluorescence staining

VSMCs were fixed with 4% paraformaldehyde for 20 min and washed twice in PBS. All slides were then permeabilized with 0.1% Triton X-100 for 5 min and blocked in 5% BSA at room temperature for 1 h. For analysis of colocalization of v-ATPase A1 and LDHB puncta, the slides were incubated with anti-v-ATPase A1 antibody (1:500) and anti-LDHB antibody (1:500) at 4 °C overnight. After the stained cells were washed with PBS, they were incubated with CoraLite488-conjugated Affinipure Goat Anti-Mouse IgG (H + L) and CoraLite594-conjugated Goat Anti-Rabbit IgG (H + L) secondary antibodies (1:2000) in the dark for 1 h at room temperature, respectively. Finally, the slides were stained with DAPI for 10 min after being washed three times in PBS and mounted with anti-fade reagent. For colocalization analysis of mitochondria and ER, cells were fixed with 4% paraformaldehyde for 20 min, washed with PBS, permeabilized with Triton as described previously, and then labelled with antibodies against TFEB (dilution 1:500; secondary antibody: CoraLite594-conjugated Goat Anti-Mouse IgG (H + L), dilution 1:2000). Slides were examined using a confocal laser scanning microscope (FV1000, Olympus). Pearson co-efficient colocalization was analysed by Image J software.

### Lysosomal acidification analysis

DND Green-189 (Yeasen Biotech, Shanghai, China) was added into cultured cells at a concentration of 1 μM and incubated at 37 °C for 30 min. After staining, cells were washed with PBS and images were taken using a confocal laser scanning microscope (FV1000, Olympus).

### Colocalization analysis of mitochondria and ER

VSMCs in each condition were stained with 200 nM of MitoTracker Green and 1 μM of ER-Tracker Red, respectively. Images were analysed using a confocal laser scanning microscope (FV1000, Olympus).

### Measurement of intracellular pyruvate level

The intracellular level of pyruvate was measured using pyruvate assay kit (Jiancheng Bioengineering Institute, Nanjing, China) according to the manufacturer’s instruction. Briefly, VSMCs seeded on six-well plates were harvested, washed twice with PBS, and lysed in RIPA buffer for 30 min. Following centrifugation for 10 min at 12,000 × *g*, the supernatants were collected. Equal volumes of substrate and cell lysate were added to each microcentrifuge tube and incubated at 37 °C for 10 min. After addition of stop solution, each sample with a volume of 200 μL was aliquoted in the wells of a 96-well microtiter plate. Pyruvate concentration was evaluated by measuring the absorbance value at a wavelength of 505 nm using a microplate reader (BIO-RAD 680, CA, USA). The result was normalized to the total protein content determined by BCA assay.

### RNA extraction and RT-qPCR

Total RNA was extracted from cultured cells by using Trizol reagent following the manufacturer’s protocol. Primers of PDK4, BMP2, RUNX2 and SM22α were designed by primer design website and were synthesized by Keygentec Biotechnology co., Ltd. (Jiangsu, China). Total RNA was reversely transcribed to cDNA using PrimeScript@RT reagent Kit and kept at −20 °C for reserve. The reaction solution was taken to carry out the fluorescence quantitative PCR, and the experiment was carried out according to the instructions of SYBR Green RT-PCR reagent. Quantitative PCR was performed using an ABI Prism 7300 sequence detection system (Applied Biosystems). The reaction conditions were as follows: pre-denaturation at 95 °C for 10 min and 40 cycles of denaturation at 95 °C for 15 s, annealing at 60 °C for 60 s and extending at 72 °C for 1 min. Relative mRNA level was analysed according to the comparative Ct method and normalized to that of GAPDH. The primer sequences used in the real-time PCR analysis were as follows: PDK4 forward: 5′-CAGACAGAGGAGGTGGTGTT-3′; PDK4 reverse: 5′-CGAGAAATTGGCAAGCCGTA-3′; BMP2 forward: 5′-AAACTTCCCGACGCTTCTTC-3′; BMP2 reverse: 5′-AGCTTCCTGCATTTGTTCCC-3′; RUNX2 forward: 5′-GATGACGTCCCCATCCATCC-3′; RUNX2 reverse: 5′-GCCAGAGGCAGAAGTCAGAG-3′; OPN forward: 5′-CTTTTGCCTGTTCGGCCTTG-3′; OPN reverse: 5′-AGTGTTTGCTGTAATGCGCC-3′; OCN forward: 5′-GGCAGCGAGGTAGTGAAGAG-3′; OCN reverse, 5′-CTGGAGAGGAGCAGAACTGG-3′; SM22α forward: 5′-CCGTGACCAAGAACGATGGA-3′; SM22α reverse: 5′-CCTCTGTTGCTGCCCATTTG-3′; GAPDH forward: 5′-GAGTCAACGGATTTGGTGGT-3′; reverse: 5′-TTGATTTTGGAGGGATCTCG-3′; LDHA forward: 5′- GCAGCCTTTTCCTTAAGACACC-3′; LDHA reverse: 5′-TCTTCCAAGCCACGTAGGTCA-3′; MCT4 forward: 5′-TATGCCTTCCCCAAAGCGGTCA-3′; MCT4 reverse: 5′-TGTCGCTGTAGCCAATCCCAA-3′; PKM2 forward: 5′-ATTGCCCGAGAGGCAGA-3′; PKM2 reverse: 5′-ACTTGGTGAGCACGATAAT-3′; GLUT1 forward: 5′-AACCGAGGGTGCCAACAA-3′; GLUT1 reverse: 5′-TGAGGTGGCTGTCGTGCAT-3′.

### Measurement of lactate production

Lactate production was determined using a lactate assay kit (Jiancheng Bioengineering Institute, Nanjing, China) according to the manufacturer’s instructions protocol. Briefly, VSMCs were harvested, rinsed twice with PBS, and lysed in RIPA buffer for 30 min. After centrifugation of the cell lysate at 12,000 × *g* for 10 min, the supernatants were collected. Equal volumes of substrate and cell lysate were added to each microcentrifuge tube and incubated at 37 °C for 10 min. Following the addition of stop solution, each sample with a volume of 200 μL were aliquoted in the wells of a 96-well microtiter plate. Lactate concentration was evaluated by measuring the absorbance value at a wavelength of 530 nm using a microplate reader (BIO-RAD 680, CA, USA). The result was normalized to the total protein content determined by BCA assay. For measurement of extracellular lactate production, the cell culture medium was collected and lactate concentration was measured with the lactate assay kit.

### Lysosomal degradation assessment

Lysosomal protease activity in VSMCs was measured using self-quenched BODIPY FL Conjugate of BSA (BioVision, USA) according to the manufacturer’s instruction. VSMCs were cultured with DMEM containing 10 µg/mL self-quenched BODIPY FL Conjugate of BSA and incubated 2 h at 37 °C. After the incubation the plate was washed twice with PBS. Lysosomal protease activity was measured using a confocal laser scanning microscope (FV1000, Olympus).

### Glucose uptake assay

Glucose uptake ability of VSMCs was evaluated by using the fluorescent glucose 2-NBDG (GlpBio, USA) according to the manufacturer’s instruction. cells were plated onto coverslips and incubated with DMEM containing 10 µM 2-NBDG at room temperature for 1 h. Then, cells were rewashed with PBS twice. Fluorescence was observed under a confocal laser scanning microscope (FV1000, Olympus).

### Lactate dehydrogenase activity

Extracellular LDH activity was detected using a commercial LDH assay kit (Jiancheng Bioengineering Institute, Nanjing, China), according to the manufacturer’s instructions.

### Seahorse XF analysis

The OCR of cells was measured using a Seahorse XF96 extracellular flux analyser (Seahorse Bioscience, USA). Briefly, cells were seeded in a 96-well Seahorse XF96 extracellular analyser plate at a density of 5000 per well to achieve 80–90% confluency at the time of assay. Basal level of OCR was recorded followed by the sequential addition of oligomycin (1 μM), FCCP (1 μM) and rotenone (0.5 μM) combined with antimycin (0.5 μM).

### Statistical analysis

Values are expressed as the mean and standard deviation (mean ± S.D.) of at least three independent experiments. Statistical analyses were performed using SPSS statistical software program 20.0 and GraphPad Prism version 7.0. Data were analysed using Student’s *t* tests (comparisons between two groups) or a one-way analysis of variance test (comparisons between multiple groups) with post hoc comparisons by Tukey’s multiple comparisons test. Differences with a *P* value <0.05 were considered statistically significant.

## Supplementary information

Supplementary Figure Legends

Supplementary Figure 1

Supplementary Figure 2

Supplementary Figure 3

Supplementary Figure 4

Supplementary Figure 5

Supplementary Figure 6

Supplementary Figure 7

Supplementary Figure 8

Supplementary Figure 9
