# Supplementary material for: PDK4 promotes vascular calcification by interfering with autophagic activity and metabolic reprogramming
Source: Cell Death Dis. 2020 Nov 17;11(11):991. doi: 10.1038/s41419-020-03162-w (PMC7673024; doi:10.1038/s41419-020-03162-w)
Supplement: Supplementary file 1 — Supplementary Figure Legends [file 41419_2020_3162_MOESM1_ESM.docx]

**Supplementary Figure Legends**

**Supplementary Fig. S1 The effects of PDK4 on the viability and proliferative capacity of VSMCs under calcification conditions.** VSMCs were transfected with lentivirus carrying shRNA targeting PDK4 or NC-shRNA for 48 h and then incubated in the presence or absence of 10 mM β-GP for 24 h. **a** Cell viability was assessed using the CCK-8 assay. N = 5 independent experiments. **b** Proliferative capacity of cells was evaluated using BrdU assay. N = 5 independent experiments. * *P* < 0.05, ** *P* < 0.01.

**Supplementary Fig. S2 Knockdown of PDK4 inhibits glycolysis.** VSMCs were transfected with lentivirus carrying shRNA targeting PDK4 or NC-shRNA for 48 h and then incubated in the presence or absence of β-GP for 72 h. **a** Protein expression of GLUT1, PKM2, LDHA, and MCT4 was measured by Western blotting. N = 3 independent experiments. ** *P* < 0.01 and *** *P* < 0.001.

**Supplementary Fig. S3 The effects of PDK4 on glucose metabolism. a** The mRNA levels of GLUT1, PKM2, LDHA, and MCT4 in aortic tissues were detected by RT-qPCR. N = 4-6 rats per group. **b** The protein expression of PKM2 and LDHA in aortic tissues was detected by Western blotting. N = 3-5 rats per group.**c** The serum lactate levels were measured with the lactate assay kit. N = 6 rats per group. * *P* < 0.05, ** *P* < 0.01, *** *P* < 0.001.

**Supplementary Fig. S4 Autophagy-related genes expression in vascular calcification.** The protein expression of LC3 and p62 in aortic tissues was detected by Western blotting. N = 3-5 rats per group. * *P* < 0.05, ** *P* < 0.01, *** *P* < 0.001.

**Supplementary Fig. S5 mTOR signaling in vascular calcification. a** VSMCs were treated with 10 mM β-GP for different timepoint. The levels of mTOR, p-TOR, and p-S6K(Thr229) were determined by Western blotting. N = 3 independent experiments. **b** VSMCs treated with RAPA in the presence or absence of β-GP. The level of p-S6K(Thr229) were determined by Western blotting. N = 3 independent experiments. * *P* < 0.05, ** *P* < 0.01, *** *P* < 0.001.

**Supplementary Fig. S6 The effects of 3-MA and RAPA on the viability and proliferative capacity of VSMCs.** VSMCs treated with either 2.5 mM 3-MA or 100 nM RAPA for 24 h. **a-b** Cell viability was assessed using the CCK-8 assay. N = 5 independent experiments. **c-d** Proliferative capacity of cells was evaluated using BrdU assay. N = 5 independent experiments. * *P* < 0.05 and ** *P* < 0.01.

**Supplementary Fig. S7 The effects of 2-DG on autophagy in VSMCs.** VSMCs treated with 100 μM 2-DG in the presence or absence of β-GP for 24 h. The protein expression of LC3 and p62 was detected by Western blotting. N = 3 independent experiments. ** *P* < 0.01, *** *P* < 0.001.

**Supplementary Fig. S8 Supplementary Fig. S7 The effects of 2-DG on necrosis in VSMCs.** Effects of 2-DG on the LDH activity in VSMCs. VSMCs were treated with 2-DG (100 µM) for 24 h and the supernatants were collected for measurement of LDH release by a commercial LDH assay kit. N = 5 independent experiments.

**Supplementary Fig. S9 The effects of lactate on the protein expression of p62.** Cells were exposed to 10 mM β-GP in the absence or presence of either lactate or 2-DG as well as DCA for 24 h. The protein expression of p62 was detected by Western blotting. N = 3 independent experiments. * *P* < 0.05, ** *P* < 0.01, *** *P* < 0.001.
