# Supplementary figures and images for: PDK4 promotes vascular calcification by interfering with autophagic activity and metabolic reprogramming
Source: Cell Death Dis. 2020 Nov 17;11(11):991. doi: 10.1038/s41419-020-03162-w (PMC7673024; doi:10.1038/s41419-020-03162-w)

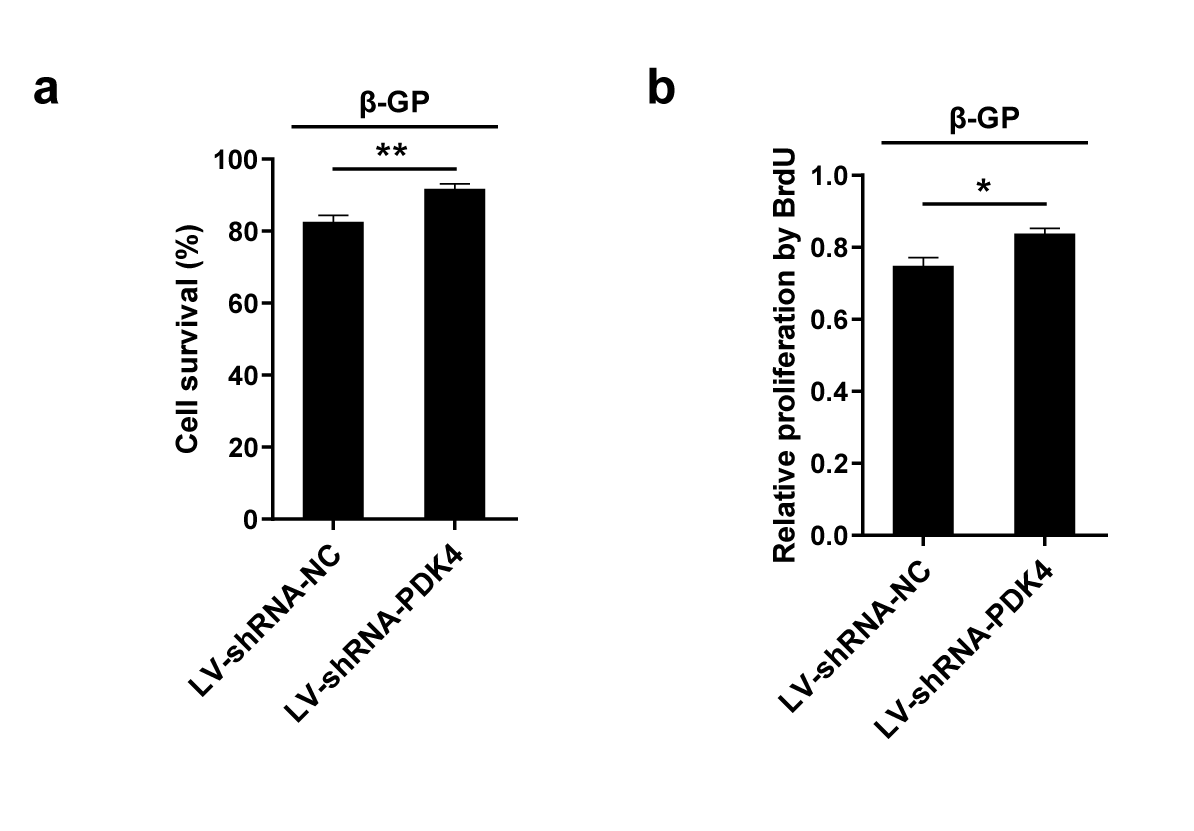

Supplement: Supplementary file 2 — Supplementary Figure 1 [file 41419_2020_3162_MOESM2_ESM.tif]

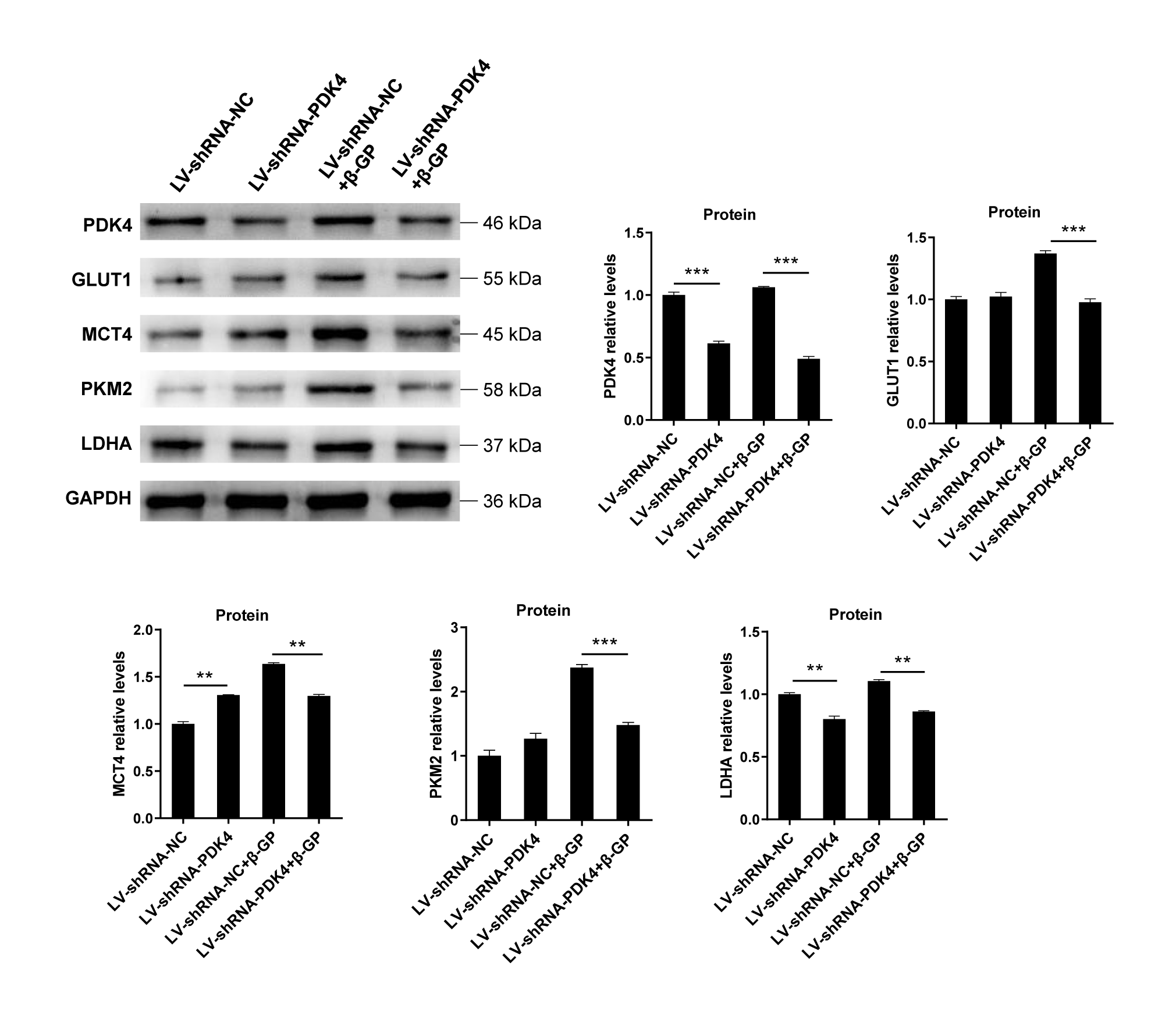

Supplement: Supplementary file 3 — Supplementary Figure 2 [file 41419_2020_3162_MOESM3_ESM.tif]

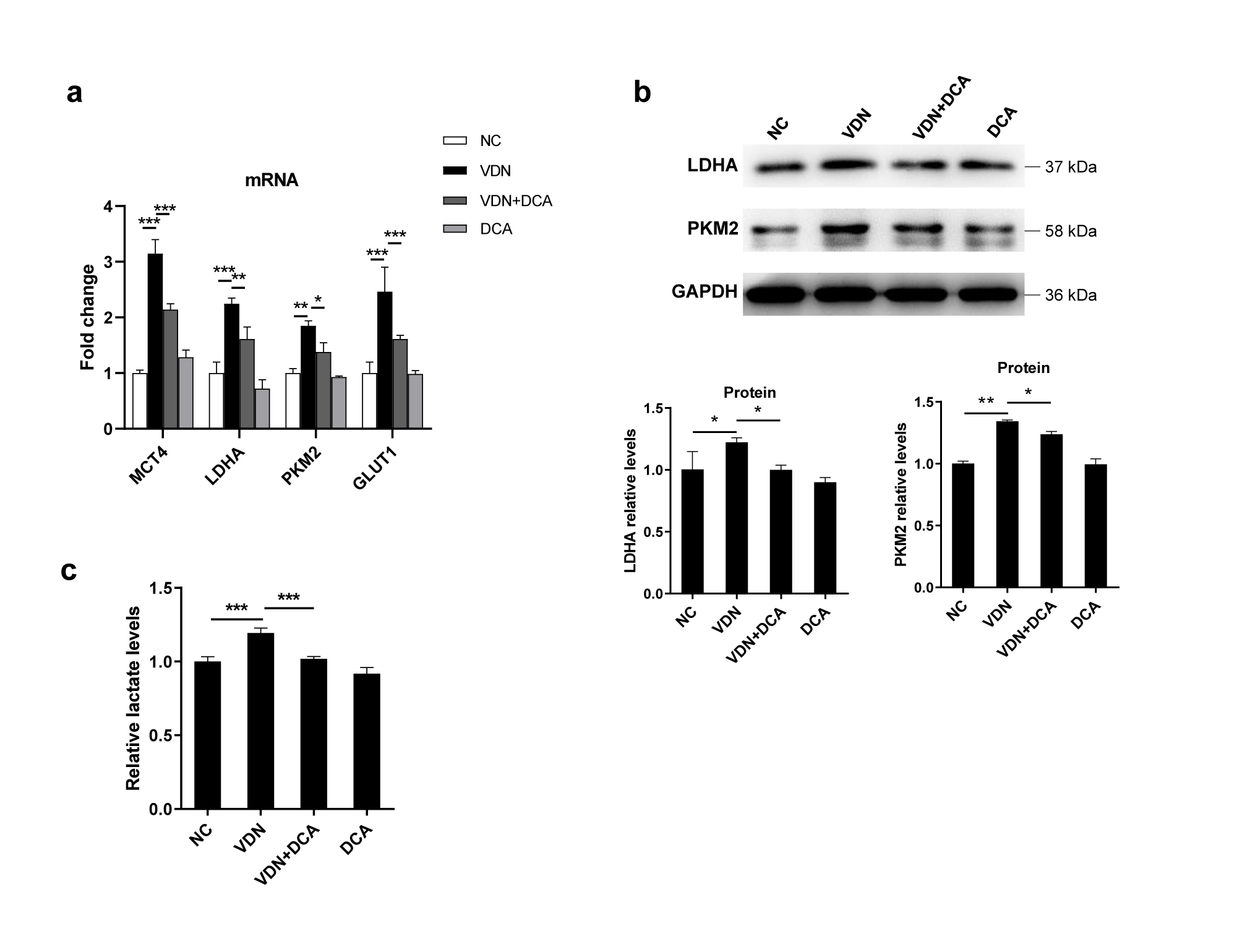

Supplement: Supplementary file 4 — Supplementary Figure 3 [file 41419_2020_3162_MOESM4_ESM.tif]

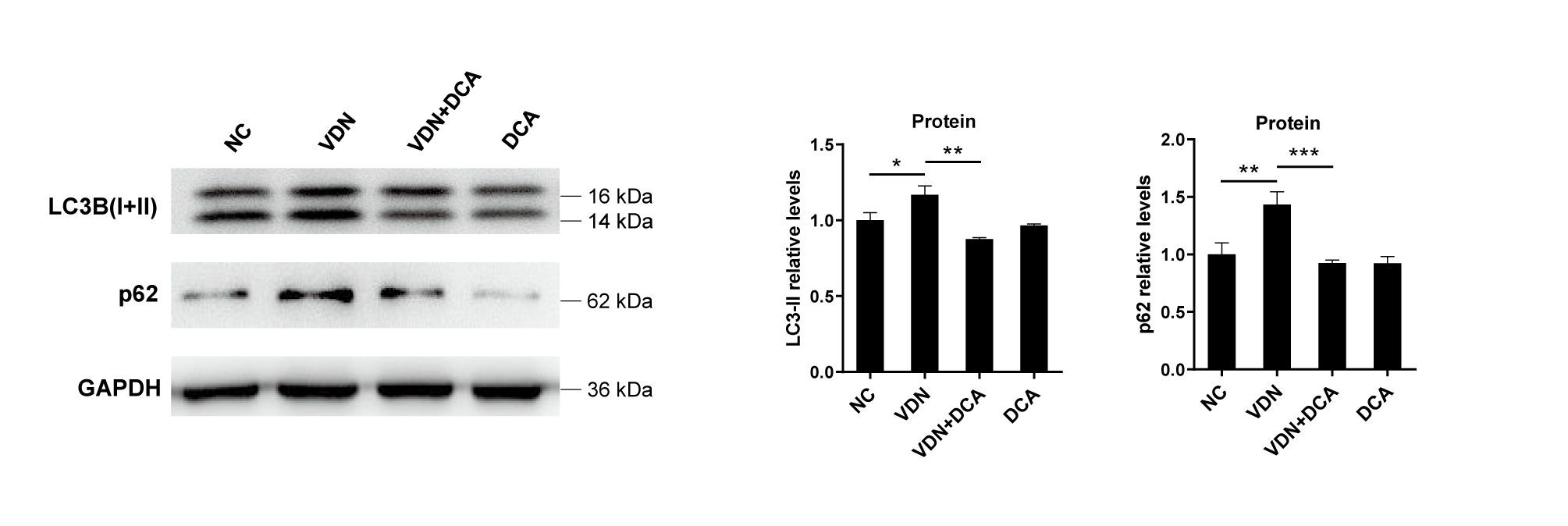

Supplement: Supplementary file 5 — Supplementary Figure 4 [file 41419_2020_3162_MOESM5_ESM.tif]

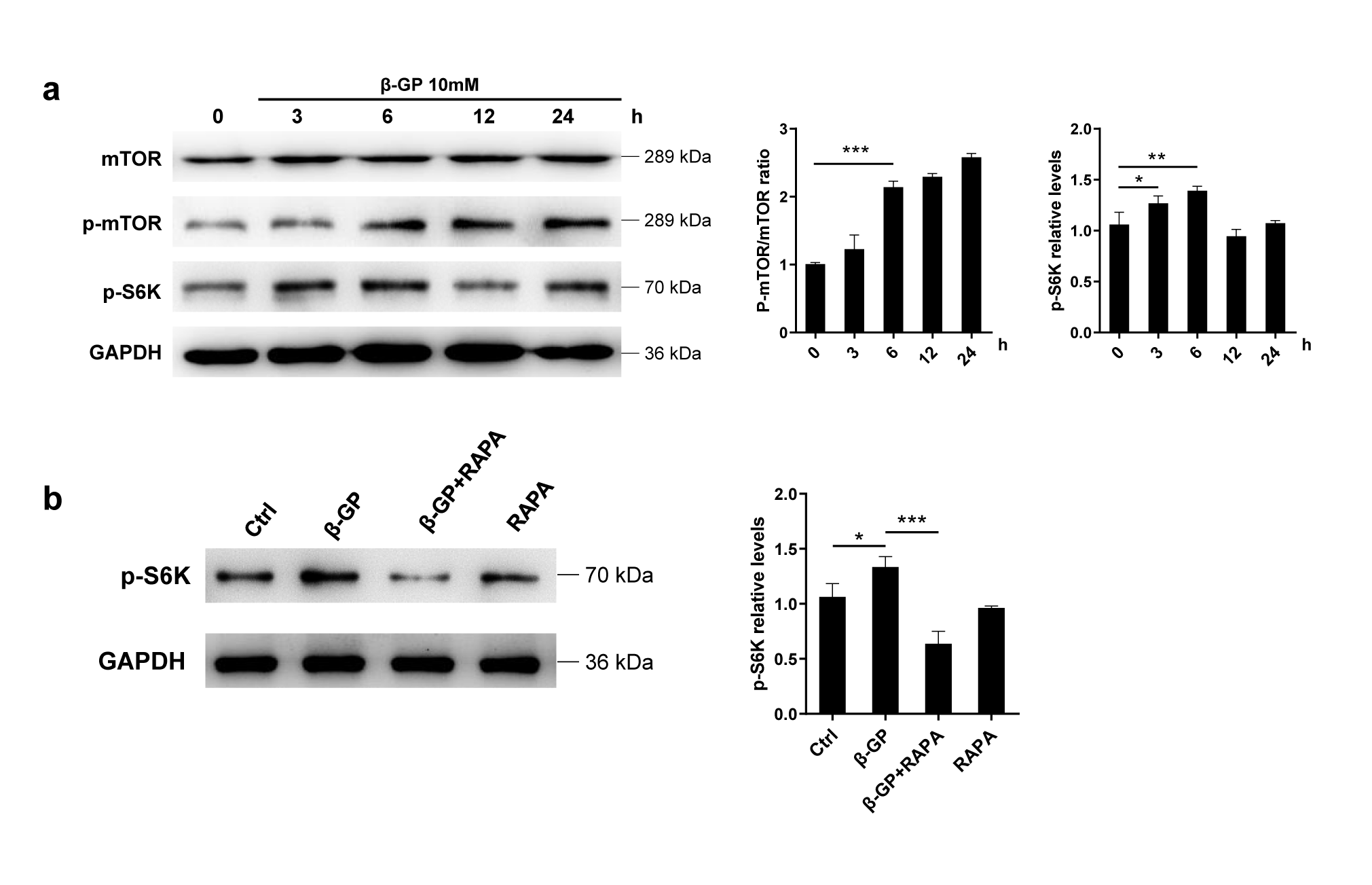

Supplement: Supplementary file 6 — Supplementary Figure 5 [file 41419_2020_3162_MOESM6_ESM.tif]

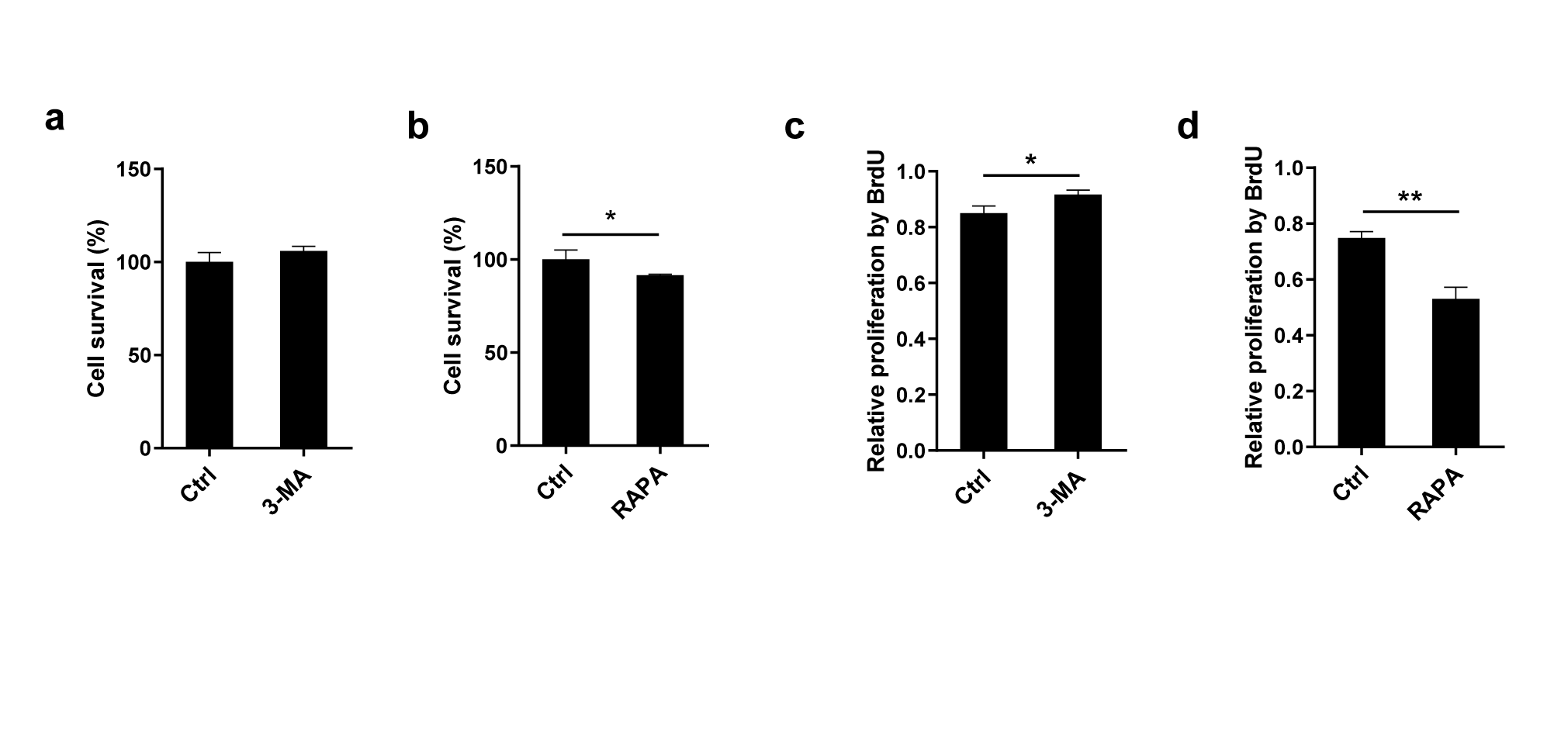

Supplement: Supplementary file 7 — Supplementary Figure 6 [file 41419_2020_3162_MOESM7_ESM.tif]

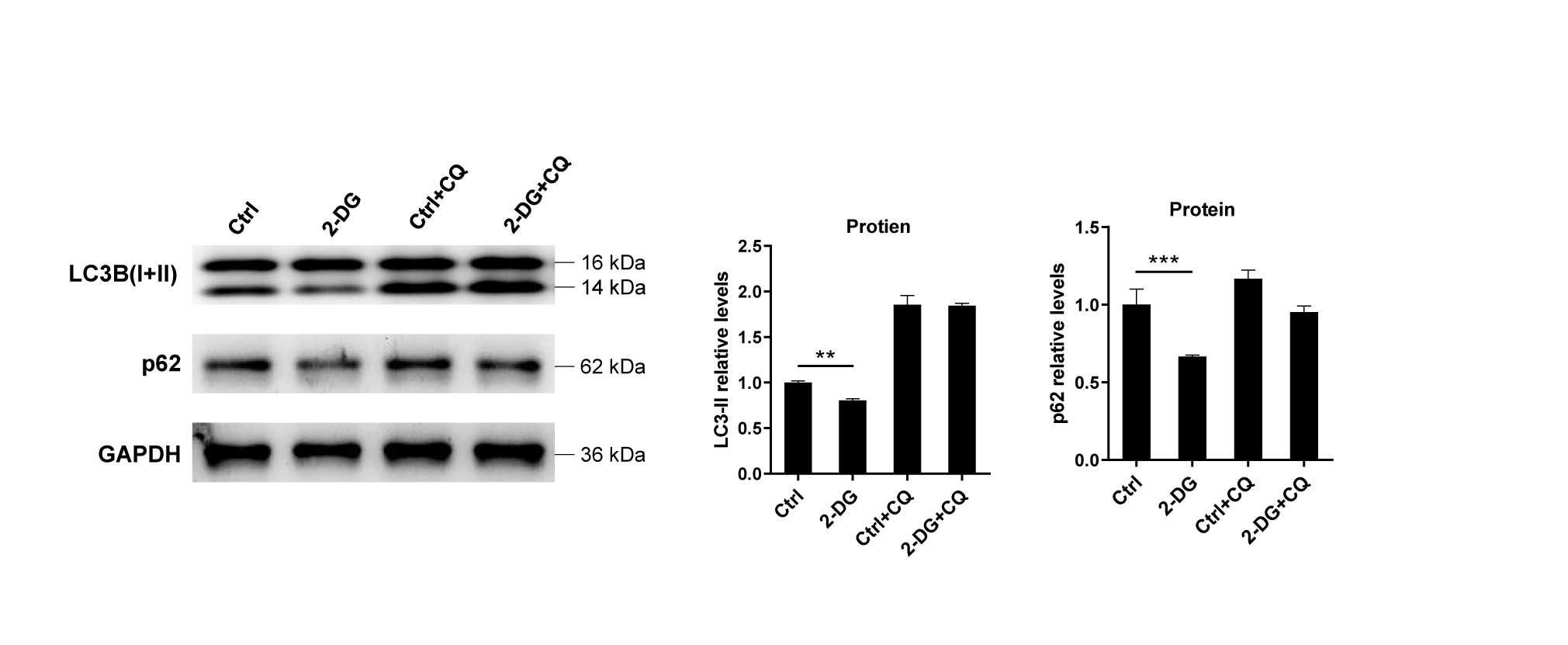

Supplement: Supplementary file 8 — Supplementary Figure 7 [file 41419_2020_3162_MOESM8_ESM.tif]

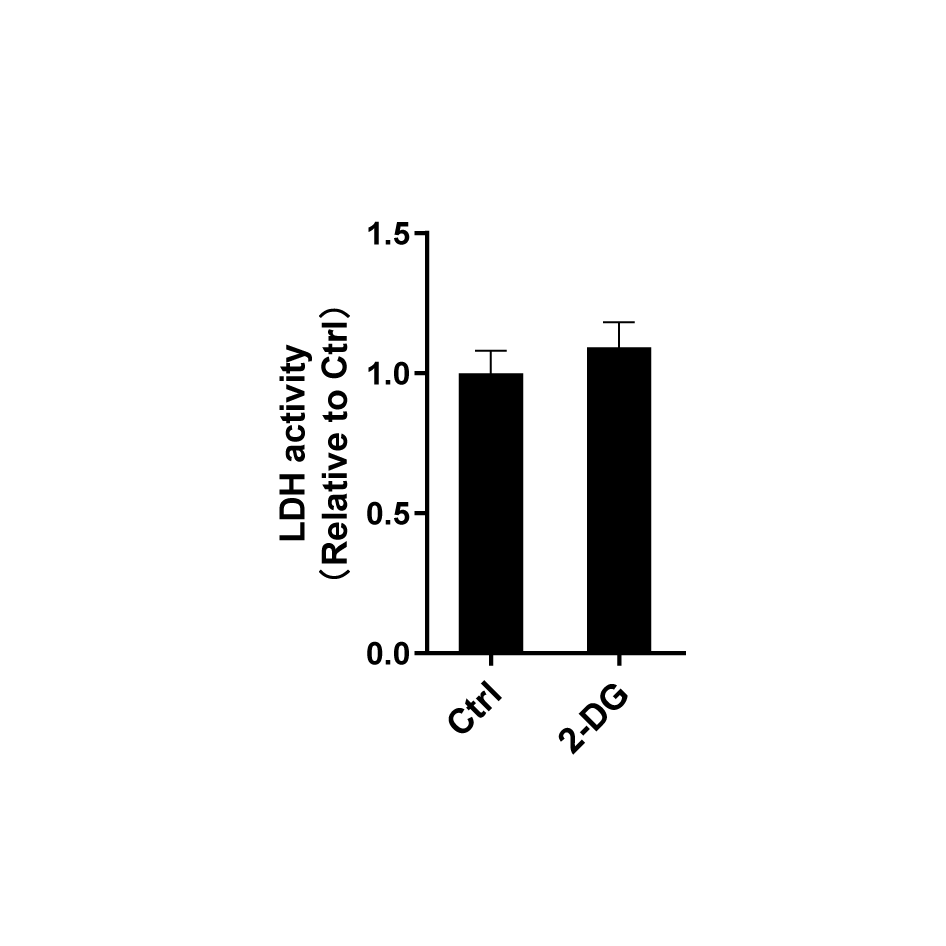

Supplement: Supplementary file 9 — Supplementary Figure 8 [file 41419_2020_3162_MOESM9_ESM.tif]

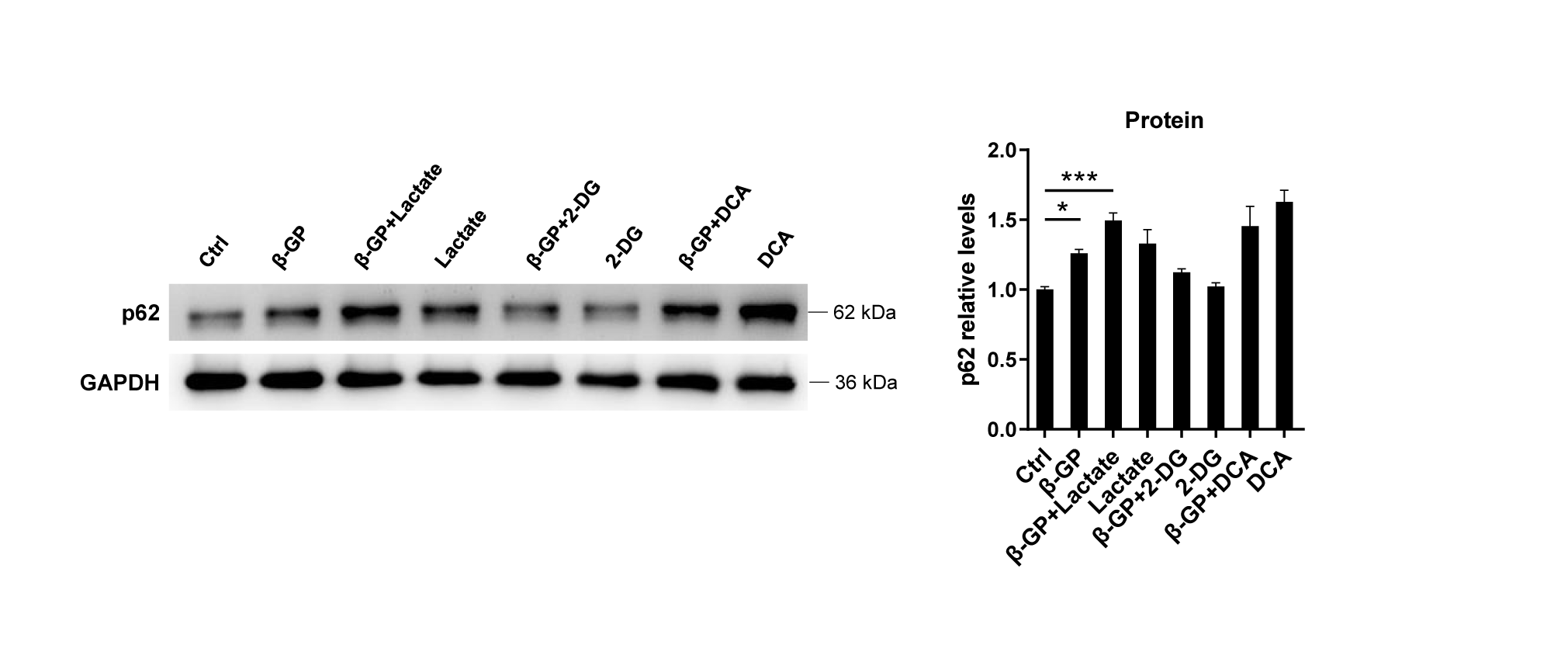

Supplement: Supplementary file 10 — Supplementary Figure 9 [file 41419_2020_3162_MOESM10_ESM.tif]
